# Time-to-Target Simplifies Optimal Control of Visuomotor Feedback Responses

**DOI:** 10.1523/ENEURO.0514-19.2020

**Published:** 2020-04-20

**Authors:** Justinas Česonis, David W. Franklin

**Affiliations:** Department of Sport and Health Sciences, Technical University of Munich, 80992 Munich, Germany

**Keywords:** motor control, optimal feedback control, reaching, time-to-target, visuomotor control, visuomotor feedback response

## Abstract

Visuomotor feedback responses vary in intensity throughout a reach, commonly explained by optimal control. Here, we show that the optimal control for a range of movements with the same goal can be simplified to a time-to-target dependent control scheme. We measure our human participants’ visuomotor responses in five reaching conditions, each with different hand or cursor kinematics. Participants only produced different feedback responses when these kinematic changes resulted in different times-to-target. We complement our experimental data with a range of finite and non-finite horizon optimal feedback control (OFC) models, finding that the model with time-to-target as one of the input parameters best replicates the experimental data. Overall, this suggests that time-to-target is a critical control parameter in online feedback control. Moreover, we propose that for a specific task and known dynamics, humans can instantly produce a control signal without any additional online computation allowing rapid response onset and close to optimal control.

## Significance Statement

Human behavior has widely been explained using stochastic optimal feedback control (OFC), formulating movement control as a set of time-dependent feedback and control gains. However, OFC is computationally expensive leading to questions about whether such a theory could be implemented in real time. Here, we show that OFC could be approximated by a simple relationship between feedback gains and the time-to-target over a variety of movement kinematics, matching the evolution of visuomotor feedback gains of our human participants during reaching. As this relationship to time-to-target is similar across a wide range of kinematics, this suggests that early stages of the OFC controlled movement could be approximated by a time-to-target control, saving computational costs and allowing for rapid execution.

## Introduction

From intercepting a basketball pass between opponents to catching a vase accidentally knocked off the shelf, visuomotor feedback responses play a familiar role in human motor behavior. Previous research has extensively analyzed these responses in human reaching movements (Day and Lyon, 2000; Saunders and Knill, 2003, 2004, 2005; Sarlegna et al., 2003; Knill et al., 2011; Reichenbach et al., 2014; de Brouwer et al., 2017, 2018), and showed an interesting combination of task-dependent variability on the timescale of a single movement (Dimitriou et al., 2013; Franklin et al., 2014, 2017; Cross et al., 2019), as well as sub-voluntary feedback onset times (Prablanc and Martin, 1992; Day and Lyon, 2000; Franklin and Wolpert, 2008; Oostwoud Wijdenes et al., 2011; Zhang et al., 2018). These visuomotor feedback responses have been shown to modulate throughout a movement depending on the perturbation onset location (Dimitriou et al., 2013). This observation was explained through optimality principles, however such control was modeled only indirectly, by replicating velocity profiles and trajectories of visually perturbed movements (Liu and Todorov, 2007; Rigoux and Guigon, 2012). In this study, we test to what degree optimal feedback control (OFC) can be used to model the visuomotor feedback responses directly.

Optimal control as a theory of human movement has normally been compared against other theories in terms of prediction of kinematics and dynamics (Todorov and Jordan, 2002; Izawa et al., 2008; Guigon et al., 2007, 2008; Nagengast et al., 2009; Yeo et al., 2016). Nevertheless, OFC has been used to motivate extensive studies investigating the control and task-dependent modulation of feedback responses (Knill et al., 2011; Pruszynski and Scott, 2012; Nashed et al., 2012, 2014). The results of these and other studies have highlighted the flexibility of the modulation of these feedback responses. While a few studies have compared the predictions of the controller feedback gains against the feedback responses in human subjects (Knill et al., 2011), such predictions have not been made about the temporal evolution of these feedback responses during reaching. For example, Dimitriou et al., 2013) show temporal evolution of feedback response intensity throughout a reaching movement, suggesting that this is similar to the feedback gain predictions of Liu and Todorov (2007). However, a direct comparison of these feedback intensities has not been made. Here, we directly compare the temporal evolution of visuomotor feedback response intensities in human participants with the prediction of these intensities in an OFC model.

Visuomotor feedback response intensity over a goal directed reaching movement follows a roughly bell-shaped profile, with peak intensity in the middle and decay toward the beginning and the end of the movement (Dimitriou et al., 2013). The results of Liu and Todorov (2007) suggest that such modulation is a combination of gains related to movement position, velocity and acceleration. However, we do not yet know whether these gains would more strongly depend on the visual kinematics or haptic kinematics. In addition, models of ball catching were shown to produce systematic errors in the prediction of the hand kinematics when using only velocity or acceleration based gains (Dessing et al., 2002), suggesting an integration of multiple state variables to produce the feedback response. Evidence of such integration then raises two important questions. First, could there be other states than position and its derivatives that also contribute to such control? Second, how can these responses be produced so rapidly, when multiple inputs need to be integrated into one solution?

One method to solve these two problems would be a controller based on time-to-target. Within a state-space system, all state variables are constantly changing with time with a fixed relationship to one another as described by the state transition and control matrices. Such a system can then be re-imagined as a system with time as its input, and these physical states as the hidden states. Such mapping simplifies the multiple input system where the inputs are state variables, to a one-input (time) system. Indeed, the expected time-to-target (or time-to-contact) has been shown to be related to the control in finger pointing (Oostwoud Wijdenes et al., 2011) and catching tasks (Dessing et al., 2002). Therefore, we test whether a simple relation to the time-to-target can explain the temporal profile of visuomotor feedback responses in humans. To test our hypotheses, we devised an experimental paradigm where we offset the usual bell-shaped velocity profile in the aim to separate the effect of the times-to-target from the effect of kinematics (both, of the hand and of the cursor) on the visuomotor feedback responses. Finally, we compare these results with a normative OFC model of visuomotor feedback responses to better understand how and whether these responses can be the result of optimality and still maintain rapid onset times.

## Materials and Methods

### Code availability

The code and the experimental data described in the paper is freely available online at https://doi.org/10.6084/m9.figshare.11323289. The code is available as Extended Data 1.

10.1523/ENEURO.0514-19.2020.ed1 Extended Data 1 All the data collected in this study, together with the analysis scripts, figure plots, and models is freely available at https://figshare.com/s/cf8df8c6e92b12742359. TTT_subdata.mat contains all the kinematics, force, and trial data of our 10 participants. The data is preprocessed: low-pass filtered as described in the methods, organized by trial and resampled at 1kHz. TTT_initialise.m loads this data and performs initial computations of variables required for some other scripts to work properly. The folder structure follows the structure of the article. The folder 1, results-basic contains all the analysis scripts that use the experimental data. The script names match the respective figure names in the article. Folders 2–7 contain various models described in the article, and scripts are named according to the relevant article figure as well. Finally, the utils folder contains the utilities for some of the previously mentioned scripts. These files have to be available when a respective simulation is being run. utils/figure_data contains exported variables for each of the figure scripts for when computations are not desired and only the figures need to be plotted. Folder simple stats contains our statistical analysis in JASP for reference. Download Extended Data 1, ZIP file.

### Participants

Eleven right-handed (Oldfield, 1971) human participants (five females; 27.3 ± 4.5 years of age) with no known neurologic diseases took part in the experiment. All participants provided written informed consent before participating. All participants except one were naive to the purpose of the study. Each participant took part in five separate experimental sessions, each of which took ∼3 h. One participant was removed from analysis as their kinematic profiles under the five experimental sessions overlapped. The study was approved by the Ethics Committee of the Medical Faculty of the Technical University of Munich.

### Experimental setup

Participants performed forward reaching movements to a target while grasping the handle of a robotic manipulandum with their right hand. Participants were seated in an adjustable chair and restrained using a four-point harness. The right arm of participants was supported on an air sled while grasping the handle of a planar robotic interface (vBOT; Howard et al., 2009). A six-axis force transducer (ATI Nano 25; ATI Industrial Automation) measured the end-point forces applied by the participant on the handle. Position and force data were sampled at 1 kHz. Visual feedback was provided in the plane of the hand via a computer monitor and a mirror system, such that this system prevented direct visual feedback of the hand and arm. The exact onset time of any visual stimulus presented to the participant was determined from the graphics card refresh signal.

**Figure 1.**
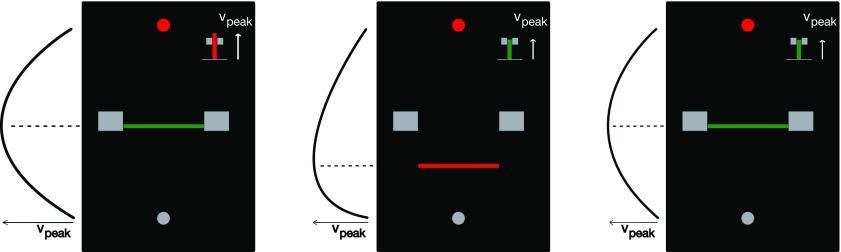
Examples of feedback presented to the participants. Feedback regarding the peak velocity and the timing of the peak velocity was provided after each trial. Large gray blocks indicate the velocity peak location target, while the bar chart at the top-right corner indicates peak *y*-velocity magnitude. Feedback was provided on the modality (cursor or hand) that matched the baseline, where the horizontal line indicated the location of the peak velocity in this modality. Left, Velocity peak location is within the target, but the movement was too fast (unsuccessful trial). Middle, Velocity peak location is too early, but the movement speed is within the target (unsuccessful trial). Right, Successful trial.

Participants initiated each trial by moving the cursor (yellow circle of 1.0 cm diameter) into the start position (gray circle of 1.6 cm diameter) located ∼25 cm in front of the participant, centered with their body. This start position turned from gray to white once the cursor was within the start position. Once the hand was within the start position for a random delay drawn from a truncated exponential distribution (1.0–2.0 s, mean 1.43 s), a go cue (short beep) was provided signaling participants to initiate a straight reaching movement to the target (red circle of 1.2 cm diameter, located 25.0 cm directly in front of the start position). If participants failed to initiate the movement within 1000 ms, the trial was aborted and restarted. Once the cursor was within 0.6 cm of the center of the target, participants were notified by the target changing color to white. The movement was considered complete when the participants maintained the cursor continuously within this 0.6 cm region for 600 ms. If participants did not complete the movement within 4 s from first arriving at the start position (e.g., by undershooting or overshooting the target), the movement timed-out and had to be repeated. Otherwise, as long as participants arrived at the target within 4 s, the trial was considered to have been completed. After each trial, the participant’s hand was passively returned by the robot to the start position while visual feedback regarding the success of the previous trial was provided (Fig. 1). Movements were self-paced, and short breaks were enforced after every 100 trials.

### Experimental paradigm

**Figure 2.**
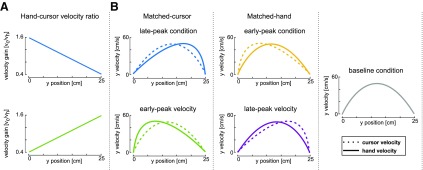
Experimental design. ***A***, top, Hand-cursor velocity scaling for conditions where the cursor position leads the hand position in *y*-axis (matched-cursor late-peak hand velocity condition, blue, and matched-hand early-peak cursor velocity condition, yellow). Bottom, Hand-cursor velocity scaling for conditions where the cursor position lags the hand position in *y*-axis (matched-cursor early-peak hand velocity condition, green, and matched-hand late-peak cursor velocity condition, purple). ***B***, Hand and cursor velocity-position profiles required to achieve the ideal movement to the target. Left, Matched-cursor velocity conditions. Middle, Baseline condition, where cursor position and hand position are consistent. Right, Matched-hand velocity conditions.

Participants performed the experiment under five different conditions, each performed in a separate session. In the baseline condition the cursor matched the forward movement of the hand, with a peak velocity in the middle of the movement. In the other four conditions, the cursor location was scaled relative to the hand location in the forward direction only (with no change in the lateral direction), such that the cursor and the hand location matched only at the start and end of the movements (Fig. 2). In two of the conditions (matched-hand velocity), the hand velocity matched the baseline condition throughout the movement (with the peak in the middle of the movement) but the cursor velocity peaked either earlier (33% of movement distance) or later (66% of movement distance). In the other two conditions (matched-cursor velocity), the cursor velocity was matched to the baseline condition throughout the movement (with the peak in the middle of the movement) but the hand velocity peaked either earlier (33% of movement distance) or later (66% of movement distance). The difference between the cursor velocity and the hand velocity was produced through a linear scaling of the cursor velocity as a function of the forward position (Fig. 2*A*). Specifically, for the two conditions where the position of the peak cursor velocity is earlier than the position of the peak hand velocity (Fig. 2, top), this scaling was implemented as:

(1) $$\frac{v_{c}}{v_{h}}=-0.012d+1.6,$$

where *v_c_* and *v_h_* are cursor and hand velocities, respectively, and *d* is the distance along the movement direction in %. The cursor velocity was therefore manipulated by a linear scaling function such that its velocity is 160% of the hand velocity at the beginning of the movement, linearly decreasing to 40% at the target location (Fig. 2, top). For the two conditions where the position of the peak cursor velocity is later than the position of the peak hand velocity (Fig. 2, bottom), this scaling was implemented as:

(2) $$\frac{v_{c}}{v_{h}}=0.012d+0.4,$$

such that the velocity gain function linearly increased from 40% hand velocity at the start of the movement to 160% at the end of the movement (Fig. 2, bottom). Desired velocity profiles of both the hand and the cursor are shown in Figure 2*B* for each condition.

Introducing the differences in velocity profiles across five experimental conditions allows us to look at the effect of the time-to-target separately from the kinematics of physical movement. First, as the visual perturbations always occurred at the five preset hand positions, our design allows us to maintain the effect of the distance to the target and distance in the movement constant across all five conditions. Second, two perturbation locations (one-third and two-thirds of movement distance) were chosen so that velocities at those locations matched across multiple conditions (early-peak condition velocity equals baseline at one-third, and late-peak velocity equals baseline at two-thirds), allowing for matching velocity contributions across conditions as well. However, across the three different physical kinematics the time-to-target is varied, allowing us to investigate whether time-to-target has an effect. Finally, the relative scaling between cursor velocity and hand velocity in the forward direction also separates the relative contributions of these two inputs, allowing us to examine the relative contributions of visual and physical kinematics in modulating the feedback responses.

### Feedback regarding movement kinematics

In all conditions, one of the velocity modalities (cursor or hand) was required to be similar to the baseline velocity profile. Feedback was always provided about the velocity modality that matched the baseline. Ideal trials were defined as trials in which this peak velocity was between 42 and 58 cm/s with the peak location between 45% and 55% of the movement distance with no target overshoot. Participants were credited one point for achieving an ideal trial and zero points otherwise, however all the trials were included in the analysis. After each trial, visual feedback about the peak velocity and the location at which this peak occurred was provided to the participants graphically (Fig. 1). The peak velocity was indicated on the right-hand side of the screen with the length of a bar and the velocity target. This bar changed color from red to green if the velocity was within the ideal range. The location of the peak velocity was indicated as a horizontal line between home and target positions at the exact location it was achieved, along with the ideal range. This line was green when the location of the peak velocity was within the ideal range, and red otherwise. Overshooting the target was defined as the position of the cursor exceeding the center of the target in the forward direction by >0.9 cm. If participants reached the target while overshooting during the movement, a message indicating the overshot was shown, no points were scored and an error tone was played to discourage further overshooting movements.

### Probe trials

**Figure 3.**
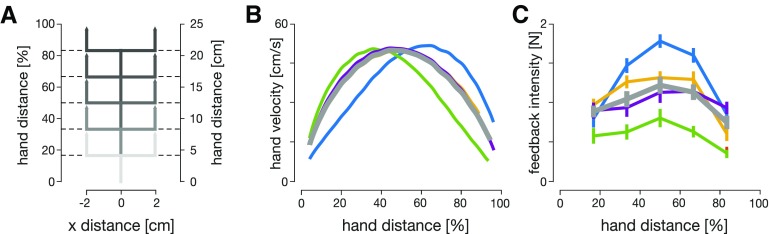
Human visuomotor feedback responses are modulated across the five experimental conditions. ***A***, Lateral perturbations of the cursor were applied in all five conditions. Perturbations were introduced as 2-cm cursor jumps perpendicular to the movement direction. The perturbation onset occurred at one of five equally spaced hand locations. ***B***, Mean velocity profiles of the hand in five experimental conditions: matched-cursor early-peak (green), matched-cursor late-peak (blue), matched-hand early-peak (yellow), matched-hand late-peak (purple), and baseline (gray). Participants successfully modulated forward movement kinematics to meet task demands, velocity profiles are skewed for matched-cursor conditions, and are similar to the baseline for matched-hand conditions. ***C***, Mean visuomotor feedback intensities (mean lateral force from 180 to 230 ms after perturbation onset) across all participants to cursor perturbations as a function of the hand distance in the movement. Error bars represent 1 standard error of the mean (SEM). Significant regulation is observed for matched-cursor early-peak and matched-cursor late-peak conditions (blue and green), but no significant regulation is seen for matched-hand conditions (yellow and purple), relative to the baseline.

During each session, probe trials were used to measure the visuomotor feedback intensity, the average strength of corrective motor response to a change in the visual feedback of hand position. To elicit these feedback responses (further visuomotor feedback responses), visual perturbations were initiated laterally (±2.0 cm) at five different hand distances (4.2, 8.3, 12.5, 16.7, and 20.8 cm) from the start (Fig. 3*A*). In addition, a zero-amplitude perturbation (cursor matched to the lateral position of the hand) was included, resulting in eleven different probe trials. On these trials the visual perturbations lasted 250 ms, after which the cursor was returned to the lateral location of the hand. The lateral hand position was constrained in these trials in a simulated mechanical channel throughout the movement, thereby requiring no correction to reach the target. The simulated mechanical channel was implemented with a stiffness of 4000 N/m and damping of 2 Ns/m acting perpendicularly to the line connecting the start position and the target (Scheidt et al., 2000; Milner and Franklin, 2005), allowing measurement of any lateral forces in response to a visual perturbation.

In previous experiments, feedback response intensity gradually decreased during the course of the experiment (Franklin and Wolpert, 2008; Franklin et al., 2012). However, it has been shown that including perturbation trials where the perturbations were maintained until the end of the movement, and where participants had to actively correct for the perturbation to reach the target, prevents this decrease in the feedback intensity (Franklin et al., 2016). Therefore, half of the trials contained the same range of perturbations as the probe trials but where these perturbations were maintained throughout the rest of the trial and participants had to correct for this perturbation. The hand movement was not constrained in these maintained perturbation trials. These maintained perturbations have now been used in several studies (Franklin et al., 2016, 2017; de Brouwer et al., 2017).

### Session design

Before each session, participants performed 100–300 training trials to learn the specific velocity profiles of the reaching movements. All training trials contained no visual perturbations and were performed in the null force field. The training trials were stopped early once participants achieved an accuracy of 75% over the last 20 trials and were not used for the analysis.

Each session consisted of 40 blocks, where each block consisted of 22 trials performed in a randomized order. Eleven of these 22 trials were probe trials (five perturbation locations × two perturbation directions + zero perturbation condition) performed in the mechanical channel. The other eleven trials consisted of the same perturbations but maintained throughout the trial and performed in the null field. Therefore, in each of the five sessions, participants performed a total 880 trials (440 probe trials). The order of the five different conditions (sessions) was pseudo-randomized and counterbalanced across participants. Participants were not told about the physical implementation of the different mappings, but were provided feedback after every trial and knew that each session was different from previous sessions.

### Data analysis

Data were analyzed in MATLAB R2017b and JASP 0.8.2. Force and kinematic time series were low-pass filtered with a tenth-order zero-phase-lag Butterworth filter (40-Hz cutoff). The cursor velocity was calculated by multiplying the hand velocity by the appropriate scaling function. The visuomotor feedback response was measured for each perturbation location as the difference between the force responses to the leftward and rightward perturbations within a block. To measure the visuomotor feedback response intensity (mean force, produced as a response to a fixed-size visual perturbation) this response was averaged over a time window of 180–230 ms, a commonly used time interval for the involuntary visuomotor feedback response (Franklin and Wolpert, 2008; Franklin et al., 2012, 2016; Dimitriou et al., 2013). In order to compare any differences across the conditions a two-way repeated-measures ANOVA was performed with main effects of condition (five levels) and perturbation location (five levels). As a secondary method to frequentist analysis we also used the Bayesian factor analysis (Raftery and Kass, 1995) to verify our statistical results. Bayesian factor analysis is a method that in addition to the conventional hypothesis testing (evaluating evidence in favor of the alternative hypothesis) allows us to evaluate evidence in favor of the null hypothesis, therefore distinguishing between the rejection of the alternative hypothesis and not enough evidence to accept the alternative hypothesis.

Although we used the time window of 180–230 ms to estimate visuomotor feedback intensity, we also verified whether the onset of the visuomotor feedback response in our data are consistent with previously reported values. To estimate this onset time, we first estimated individual onset times for each participant at each perturbation location and movement condition. To do so, we used the receiver operator characteristic (ROC) to estimate where the force reaction to leftwards cursor perturbations deviated from the reaction to rightwards cursor perturbations (Pruszynski et al., 2008). For each type of trials, we built the ROC curve for the two signals at 1 ms intervals, starting from 50 ms before the perturbation, and calculated the area under this curve (aROC) for each of these points until the aROC exceeded 0.75 for 10 consecutive milliseconds. In order to find where the force traces start deviating from each other, we then fit a function of the form max(0.5,k×(t−τ) to the aROC curve. The time point where the linear component of this function first overtakes the constant component was taken as the threshold value. Overall, the mean onset times across all conditions and perturbation locations were 138 ± 7 ms (mean ± SD), with onset times consistent among movement conditions (*F*_(4,36)_ = 1.410, *p *=* *0.25, and *BF*_10_ = 0.105), perturbation locations (*F*_(4,36)_ = 1.582, *p *=* *0.20, *BF*_10_ = 0.252), and their interactions (*F*_(16,144)_ = 1.350, *p *=* *0.176, and *BF*_10_ = 0.005)

### Modeling

#### OFC

In addition to our linear models we implemented two different OFC models: the classical model (Liu and Todorov, 2007) and the time-to-target model. The only comparison between the output of the optimal control models and the experimental results is via the feedback gains. For each movement we define time-to-target as the duration between the onset of the perturbation and the cursor first intercepting the target. In both models we modeled the hand as a point mass of *m *=* *1.1 kg and the intrinsic muscle damping as a viscosity *b *=* *7 Ns/m. This point mass was controlled in a horizontal plane by two orthogonal force actuators to simulate muscles. These actuators were controlled by the control signal *u_t_* via a first order low-pass filter with a time constant *τ* = 0.05 s. The state-space representation of the dynamic system used to simulate the reaching movements can be expressed as

(3) $$x_{t+1}=Ax_{t}+B(I+C)u_{t}+\xi_{t},$$

where *A* is a state transition matrix, *B* is a control matrix, and C is a 2 × 2 matrix, whose each element is a zero-mean normal distribution representing control-dependent noise. Variables *x_t_* and *u_t_* are state and control at time *t*, respectively. State *x_t_* exists in the Cartesian plane and consists of position $\vec{p}$ (2 dimensions), velocity $\vec{v}$ (2), force $\vec{f}$ (2), and target position $\vec{p^{*}}$ (2). The presence of these four states within the state vector means that the information about all of these states is eventually used for the control. For our simulation purposes we treat the control-independent noise *ξ_t_* as zero.

The state of the plant is not directly observable, but has to be estimated from noisy sensory information. We model the observer as:

(4) $$y_{t}=Hx_{t}+D_{t},$$

where H=diag[1,1,1,1,1,1,0,0] is the observation matrix, and *D_t_* is a diagonal matrix of zero-mean normal distributions representing state-independent observation noise. Therefore, our observer can infer the state information of position, velocity and applied force of the plant, consistent with human participants.

The simulated movements were guided by the LQG controller with a state-dependent cost Q, an activation cost R, a reaching time N, and a time step *t* = 0.01 s. However, due to the presence of the control-dependent noise, the estimation and control processes are not anymore separable as in the classic LQG theory. In order to obtain optimal control and Kalman gain matrices we used the algorithm proposed by Todorov and Li (2005), where control and Kalman gain matrices are iteratively updated until convergence.

For both the classical and time-to-target models we simulated three different movement kinematics representing three different conditions in our experiment, the baseline and the two matched-cursor conditions. The state-dependent cost *Q* was identical for all three kinematics:

(5) $$Q(t)=\{\begin{array}{l} 0,\;\text{for}\;t\neq N\\ {\left(\vec{\omega_{p}}(\vec{p}(t)-\vec{p^{*}}(t))\right)}^{2}+\omega_{v}||\vec{v}(t)|{|}^{2}+\omega_{f}||\vec{f}(t)|{|}^{2},\;\text{for}\;t=N \end{array},$$

where $\vec{\omega_{p}}=[0.5,1],\;\omega_{v}=0.02$, and *ω_f_* = 2. The activation cost R(*t*)* *=* *0.00001 was constant throughout the movement for the baseline condition, but was modulated for the two matched-cursor conditions by multiplying it elementwise by a scaling function:

(6) $$R\prime (t)=\frac{\exp(p\frac{t+q}{r})}{mean(R\prime )},$$

where *p*, *q*, and *r* are constants.

Thus, each movement condition only differed from the other two by the profile of this activation cost R, but not by its magnitude. These modified activation costs shift the timing of the peak velocity toward either the beginning or the end of the movement by penalizing higher activations at either the end or beginning of the movements, respectively.

The mean activation cost is kept constant across the conditions resulting in each condition being equally “effortful.” All other simulation parameters were kept constant across the three conditions.

Although LQG is a fixed time horizon problem, we did not predefine the movement duration N. Instead, we obtained the N, and constants *p*, *q*, and *r* using Bayesian adaptive direct search (BADS; Acerbi and Ma, 2017) to maximize the log-likelihood of the desired peak velocity location and magnitude. We did not fit any other parameters beyond this point. Rather, we analyzed our models’ qualitative behavior compared with human participant data.

The classical and the time-to-target models only differed in the way the perturbations were handled. For the classical model, we simulated perturbation trials at every time step *t_p_* by shifting the target *x*-coordinate by 2 cm at the time *t_p_* + 120 ms. This 120 ms delay was used to mimic the visuomotor delay in human participants, and was taken from Liu and Todorov (2007). We then averaged the force response of the controller over the time window [*t_p_*+130, *t_p_*+180] as an estimate of the simulated feedback responses, equivalent of visuomotor feedback responses in our participants. This means that our simulated feedback responses arise due to separate contributions from the controller position, velocity and acceleration gains. For perturbations occurring at times where the movement is over before the end of this time window, the intensity of this simulated feedback response is set to zero.

For the time-to-target model we introduced an extension in the time-to-target after the onset of any perturbation similar to that observed in our participants. Simulated feedback intensities were modeled at five locations, matching the perturbation locations in our experiment to obtain the appropriate increase in time-to-target after each perturbation. In order to simulate the response to perturbations we first extracted the perturbation onset times from movement kinematics by performing an unperturbed movement and recording the time point *t_p_* at which this movement passed the perturbation onset location. We then simulated the postperturbation portion of the movement as a new LQG movement with an initial state matching the state at *t_p_* + 120 ms of the unperturbed movement, and movement duration matching the time-to-target recorded in our participants for the particular perturbation. Therefore, our time-to-target model can only simulate the feedback intensities at the five perturbation locations in the movement. Together, this keeps our simulated reaches “naive” to the perturbation before its onset and allows the time-to-target of the simulated reaches to match the respective time-to-target of our human participants. Finally, we calculated the simulated feedback intensities as described previously, using a time window [10, 60 ms] of the postperturbation movement. As in the previous simulations, these simulated feedback responses arise due to separate contributions from the controller position, velocity and acceleration gains.

#### Time-to-target tuning function

In order to understand the mechanisms that might underlie the consistent relationship between the simulated feedback intensities and the time-to-target, we fit a mathematical expression to the simulated feedback intensities. We modeled the relationship as the minimum of a squared-hyperbolic function and a logistic function:

(7) $$G(t)=\min(\frac{\beta }{{\left(t-t_{1}\right)}^{2}};\;\frac{\alpha }{1+\exp(-\frac{t-t_{0}}{\tau })}),$$

and used BADS to fit this function to our time-to-target-simulated feedback intensity data by optimizing the log-likelihood of this fit.

While the logistic function was chosen simply as it provided a good fit to the data, the squared-hyperbolic arises from the physics of the system. Specifically, from the kinematic equations of motion for a point mass (*m*) traveling a distance (*d*) under the influence of force *F*, the distance can be expressed as:

(8) $$d=\frac{Ft^{2}}{2m}+v_{0}t,$$

where *v*_0_ = 0 is the lateral velocity at the start of perturbation correction. Rearranging gives:

(9) $$F=\frac{2md}{t^{2}}\propto \frac{1}{t^{2}}.$$

Hence the lateral force necessary to bring a point mass to the target is proportional to $1/t^{2}$.

#### Receding horizon OFC

In addition to our finite horizon control we also implemented a receding horizon controller (Guigon et al., 2019). Irrespectively of the current state of the movement *X_t_*, the receding horizon controller is defined to aim to arrive at the target at time *t* + *T_h_*. In essence, such controller is therefore not different from the finite horizon controller in its implementation for a single state of the movement. We implemented the receding horizon controller by iterating a finite horizon controller described previously, but with the *T_h_* = 500 ms, and Q and R costs scaled from the finite horizon model to fit the movement duration. For each iteration we recorded the next movement state (10 ms away from the initial state), and used that as the initial state for the next iteration. This process was repeated until the cursor was within the distance of 0.4 cm from the target position, and remained there without overshooting for 600 ms.

Simulating differently skewed velocity profiles within the framework of receding-horizon control is non-trivial. As a result, we chose to only model one, the baseline, experimental condition, where the activation cost R is constant within the movement. Therefore, we chose the costs

(10) $$Q(t)=\{\begin{array}{l} 0,\;\text{for}\;t\neq T_{h}\\ \vec{\omega_{p}}{\left(\vec{p}(t)-\vec{p^{*}}(t)\right)}^{2}+\omega_{v}||\vec{v}(t)|{|}^{2}+\omega_{f}||\vec{f}(t)|{|}^{2},\;\text{for}\;t=T_{h} \end{array},$$

where $\vec{\omega_{p}}=[5,5],\;\omega_{v}=0.05$, and *ω_f_* = 5. and the activation cost R = 0.000003. The values were selected so that the movement durations, produced by the receding-horizon model would match the experimental durations for the baseline condition. However, the resultant velocity profiles of this model more closely resembled those of the early-peak velocity condition, than those of the baseline. To account for any effects of the velocity profile we also fit the costs so the model prediction of movement durations matched the durations of the early-peak velocity condition. For this simulation we selected $\vec{\omega_{p}}=[0.7,0.7],\;\omega_{v}=0.007$, and *ω_f_* = 0.7, while the activation cost remained unchanged.

In this model we introduced the simulated perturbation by shifting the target position by 2 cm at 120 ms after the *y*-coordinate of the movement passed the perturbation onset location. We only simulated the perturbations matching our experimental conditions, lateral 2 cm cursor jumps, with the onset at five evenly distributed forward distances. We calculated simulated feedback intensities the same way as for the classical and time-to-target models.

#### Infinite horizon OFC

We implemented the infinite horizon OFC to control our simulated hand based on the previous work of Qian et al. (2013). Specifically, we calculated the control gain matrix L, and Kalman gain matrix K to control the same system as in the previous models. We chose the state-dependent costs $\vec{\omega_{p}}=[1,1],\;\omega_{v}=0.02$, and *ω_f_* = 0 for the baseline condition simulation, and $\vec{\omega_{p}}=[0.35,0.35],\;\omega_{v}=0.007$, and *ω_f_* = 0 for the early-peak condition simulation. For both conditions, the activation cost R = 0.002 was kept the same. The protocol of simulating the mean trajectories, feedback responses and their intensities was otherwise identical to the receding horizon simulations.

#### Model comparison

We compared the simulated feedback intensities from each of the models with the experimental feedback responses intensities to evaluate our models. We do not evaluate models in terms of kinematics or any other variables. As the predictive simulated feedback intensities for each of the four models provided very different patterns, the important comparison is qualitative. However, we supplemented this qualitative comparison with a quantitative model comparison using the Bayesian information criterion (BIC). BIC is a conventional method for model comparison which evaluates the log-likelihood of the model fitting to the data while controlling for over-fitting by penalizing additional model parameters (Schwarz, 1978). A BIC difference of 10 is very strong evidence for the model with the lower BIC. Overall, we used individual participant mean feedback intensities for baseline, early-peak hand velocity and late-peak hand velocity conditions, providing us with a total of 150 data points (10 participants × three conditions × five perturbations) to determine the fit. Moreover, to compare the captured variance of the data between our OFC models and the time-to-target tuning curve we also calculated the sum of squared-residuals (SSRs) between the models and the data.

## Results

### Experimental results

In this study, we examine the relation between time-to-target (the time difference between the perturbation onset and the cursor intercepting the target) and the visuomotor feedback responses. To do so, we devised an experiment consisting of five different kinematic conditions. The baseline condition required movements with a natural, bell-shaped velocity profile, while the velocity profiles were modified for the four other conditions. In these four conditions, we introduced a manipulation between the hand velocity and the cursor velocity in the forward direction, such that the cursor and hand had different velocity profiles, but their positions matched at the start and end of the movement (Fig. 2). Two of these four conditions (matched-cursor conditions) required different kinematics of the physical movement to successfully complete the task, but the cursor velocity profiles matched the baseline. This manipulation of hand velocity profiles also resulted in different times-to-target at the same distance in the movement. The two other conditions (matched-hand conditions) required the same hand movement as for the baseline condition, but as a result the cursor moved with different velocity profiles (see Materials and Methods). This manipulation of the cursor velocity profiles separates the relative contributions of physical and visual hand information in regulating the feedback responses. For each condition we measured the visuomotor feedback intensities (mean corrective force applied during the 180 to 230 ms time window after a visual perturbation) at five different locations in the movement (Fig. 3*A*). Overall, our paradigm allowed us to modulate the times-to-target across conditions, as well as separate proprioceptive (hand) and visual (cursor) kinematics to examine their individual contribution to visuomotor feedback responses.

Different movement conditions exhibited differences in visuomotor feedback intensities (Fig. 3). Two-way repeated-measures ANOVA (both frequentist and Bayesian; Materials and Methods) showed significant main effects for both condition (*F*_(4,36)_ = 10.807, *p *<* *0.001, and $BF_{10}=9.136\times 10^{12}$), and perturbation location (*F*_(4,36)_ = 33.928, *p *<* *0.001, and $BF_{10}=6.870\times 10^{9}$). *Post hoc* analysis on movement conditions revealed significant differences between baseline (gray line) and matched-cursor late-peak hand velocity condition (blue line; *t*_(9)_ = 4.262, *p_bonf_* < 0.001 and *BF*_10_ = 247.868), and between baseline and matched-cursor early-peak hand velocity condition (green line; *t*_(9)_ = –8.287, *p_bonf_* < 0.001 and *BF*_10_ = 1.425 × 108). However, no significant differences were found between the baseline and the two matched hand velocity conditions (*t*_(9)_ = 1.342, *p_bonf_* = 1.0 and *BF*_10_ = 0.357 for early-peak cursor velocity, yellow; *t*_(9)_ = 0.025, *p_bonf_* = 1.0 and *BF*_10_ = 0.154 for late-peak cursor velocity, purple). Our results show that different kinematics of the hand movement have a significant effect on visuomotor feedback response regulation, but that different kinematics of the cursor movement do not.

One possible explanation for differences between the two matched-cursor conditions (Fig. 3*C*, blue and green) and the baseline condition (gray) might arise from a different mapping between cursor and hand velocities (Fig. 2*A*) that had to be learned. Alternatively, the incongruency between the vision and proprioception might be another explanation. However, the two matched-hand conditions (yellow and purple) had the identical mappings (and incongruencies) as the two matched-cursor conditions (blue and green, respectively) and yet no differences were found in these conditions. Instead, the only conditions in which differences in the feedback gains were found, were conditions in which the timing of the peak hand velocity was shifted.

**Figure 4.**
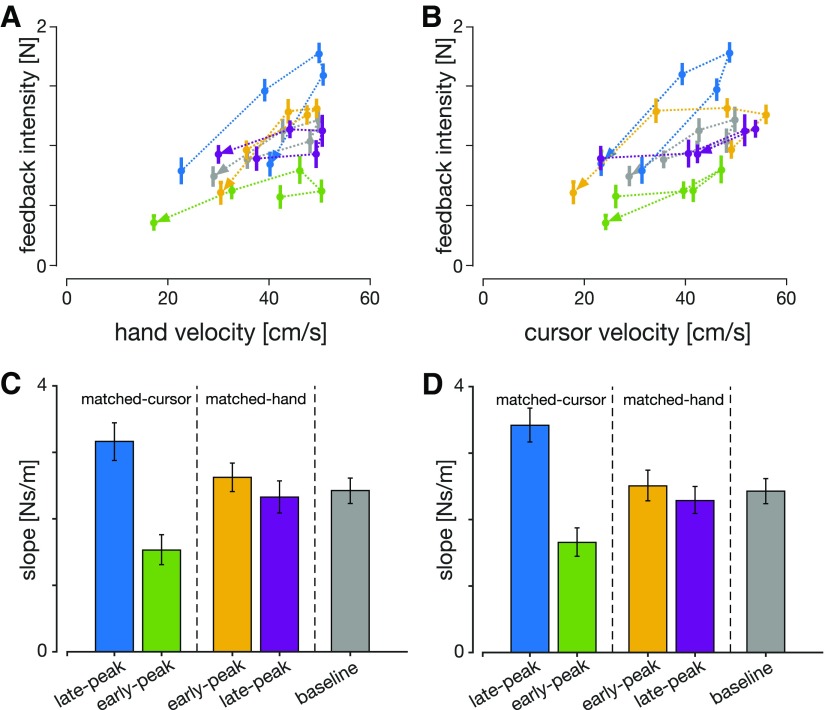
Visuomotor feedback intensities as a function of (***A***) hand velocity and (***B***) cursor velocity at the time of perturbation for all experimental conditions. Error bars represent 1 SEM, and the arrowheads represent the order of the perturbation locations. ***C***, ***D***, Regression slopes of feedback intensities for each condition as a function of hand and cursor velocities, respectively. Error bars represent 95% confidence intervals of the slopes. The slopes for the two matched-cursor conditions were significantly different (based on the confidence intervals) than for the baseline condition.

In order to test whether a simple relationship between movement kinematics and visuomotor feedback intensities exists, we mapped visuomotor feedback intensity magnitudes as a linear function of the hand velocity and the cursor velocity. For each experimental condition, we find a different regression slope between the velocity and the feedback intensities regardless of whether this is the cursor or the hand velocity (Fig. 4*A*,*B*). Consistent with our previous results, this difference in slopes is significant for conditions where the hand, but not cursor, movement was different (Fig. 4*C*,*D*). Although feedback intensities increase with increasing velocity in both cursor and hand coordinates, no one coordinate modality could predict the changes in the feedback intensity.

**Figure 5.**
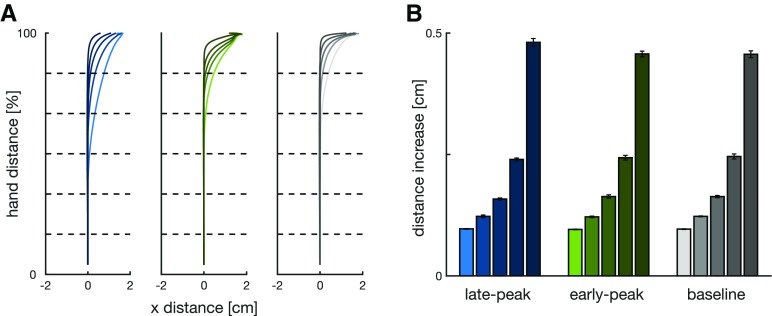
***A***, Mean hand movement trajectories for matched-cursor late-peak (left), matched-cursor early-peak (middle), and baseline (right) conditions recorded in our participants, with perturbation onset at five locations [color light to dark: 4.2 cm (16.7%), 8.3 cm (33.3%), 12.5 cm (50%), 16.7 cm (66.7%), and 20.8 cm (83.4%) from the start position; dashed lines]. Corrections to rightward perturbations were flipped and combined with leftward corrections. ***B***, Distance increase for each perturbation location recorded in our participants. Perturbation locations closest to the target required the largest increases in movement distance. Error bars represent 1 SEM.

**Figure 6.**
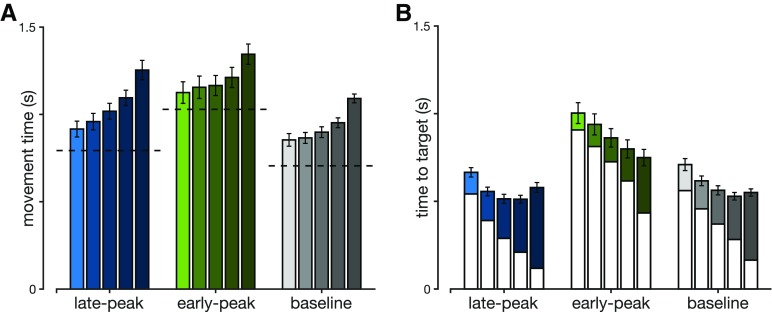
***A***, Movement durations in maintained perturbation trials recorded by our participants in late-peak, early-peak and baseline conditions. Separate bars within the same color block represent different perturbation onset locations (left to right: 4.2, 8.3, 12.5, 16.7, and 20.8 cm from the start position). Error bars represent 1 SEM while the horizontal dashed lines represent movement durations in the same movement condition for non-perturbed movements. ***B***, Full bars represent times-to-target (time between a perturbation onset and target interception) in maintained perturbation trials in our participants for late-peak, early-peak, and baseline conditions. White bars represent the time-to-target for a respective non-perturbed movement, at the time when the perturbation would have happened. The colored part of the bars represents the extension in times-to-target due to the perturbation in a non-constrained movement. This shows that the perturbation during the movement evokes an extension in the time-to-target and subsequently in movement duration Each of the five bars represents a different perturbation onset location, as in ***A***. Error bars represent 1 SEM.

To successfully complete each trial, participants were required to reach the target. However, the distance to reach the target is affected by the perturbation onset, later perturbation locations lead to larger correction angles (Fig. 5*A*) and thus longer movement distances (Fig. 5*B*). That is, an earlier correction means that the trajectory can go directly toward the target, whereas a later correction would require a new corrective movement and therefore further distance. This effect is clearly seen where the extension of movement distance is enhanced for the perturbations closest to the target, with movement distance extended by almost 0.5 cm compared with less than 1 mm for the closest perturbations. Any extension of the movement distance requires an appropriate increase in movement duration. Consequently, participants extended their movement time, with longest durations for perturbations close to the target (Fig. 6*A*). This increase in movement duration increases the time-to-target for these late perturbations (Fig. 6*B*), and now allows sufficient time for the controller to issue any corrective commands.

### Finite horizon OFC

As optimal control has been suggested to predict the temporal evolution of feedback intensities (Liu and Todorov, 2007; Dimitriou et al., 2013), we built two finite-horizon OFC models: the classical model (Liu and Todorov, 2007), and a time-to-target model. For the classical model we implemented an OFC (Todorov, 2005) to simulate movements with different velocity profiles, similar to the experiments performed by our participants. We extended this classical model to the time-to-target model, by increasing the movement duration after each perturbation onset according to experimental results (Fig. 6). For both models we only simulated different hand kinematics for computational ease and as our participants showed little effect of cursor kinematics on their feedback intensities.

**Figure 7.**
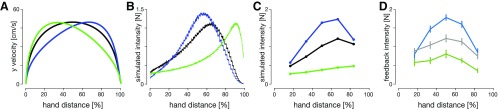
Comparison of feedback intensities between the two OFC models and experimental data. Simulated velocity profiles (***A***) and simulated feedback intensity profiles (***B***) of baseline (black), early-peak (green), and late-peak (blue) velocity condition simulations for the classical OFC model. Velocity profiles were obtained by constraining the velocity peak location and magnitude and optimizing for movement duration and activation cost function. Simulated feedback intensity profiles were obtained by applying virtual target jumps perpendicular to the movement direction during these movements and calculating the force exerted by the controller in the direction of the target jumps. The jagged appearance of the intensity traces is simply an outcome due to the simulation time step. ***C***, Simulated feedback intensities obtained via the time-to-target OFC model. Preperturbation movements were simulated as if no perturbation would occur, to keep the controller naive to an upcoming perturbation. At the perturbation onset the remaining movement duration is adjusted to match the mean time-to-target for a similar perturbation onset in human participants (Fig. 6*B*). Therefore, this model only simulates the feedback intensities at the five perturbation locations in the movement. The velocity profiles for the time-to-target model match the velocity profiles of the classical model, shown in ***A***. ***D***, Visuomotor feedback intensities recorded in human participants.

For both models we controlled the activation cost R to simulate three conditions in which the location of the peak velocity was shifted to match the experimental hand kinematics (Fig. 7*A*). Specifically, we solved for the activation cost R and movement duration N by optimizing the log-likelihood of our model’s peak velocity location and magnitude using BADS (Acerbi and Ma, 2017). The optimized movement durations (mean ± SEM) were N = 930 ± 0 ms for the baseline condition, N = 1050 ± 10 ms for the late-peak condition and N = 1130 ± 20 ms for the early-peak condition (10 optimization runs per condition). In comparison, experimental movement durations were N = 932 ± 30 ms for the baseline condition, N = 1048 ± 47 ms for the late-peak condition and 1201 ± 59 ms for the early-peak condition, matching well with the OFC predictions. Overall, this shows that specific constraints on the magnitude and location of peak velocity that we imposed on our participants resulted in a modulation of reaching times that matched OFC predictions under the same constraints.

For the classical model we estimated simulated feedback intensities by shifting the movement target at each time point in the movement and measuring the mean magnitude of the simulated force response over a 130- to 180-ms time window in the direction of this shift. The simulated feedback intensity profiles follow the same general shape as in human participants, intensity increases from the beginning of the movement and then falls off at the end (Fig. 7*B*). However, the overall profile of these simulated feedback intensities is very different for each of the kinematic conditions. For the early-peak velocity condition, the simulated feedback intensity peaks toward the end of the movement (green line), whereas for the late-peak velocity condition the simulated feedback intensity profile peaks early in the movement (blue line). These simulated feedback intensities do not appropriately capture the modulation of visuomotor feedback intensities in our experimental results. Specifically, they predict a temporal shift in the peak intensity that is not present in our participants data, and predict similar peak levels of feedback intensities across all three conditions. While the simulated feedback intensities are qualitatively similar to the experimental results within each condition; overall, this model cannot appropriately capture the modulation of visuomotor feedback responses across the conditions.

For the time-to-target OFC model, we extended the classical model to account for the different movement durations for each perturbation location (and movement condition) that is seen in the experimental results. After a perturbation, the remaining time-to-target was adjusted to match the experimentally recorded times-to-target for this specific movement, while before the perturbation both the classical model and the time-to-target model were identical. After adjusting for the individual durations of each perturbation condition we are now able to qualitatively replicate the general regulation of feedback intensity profiles for different kinematics using OFC (Fig. 7*C*). In the late-velocity peak condition we predict a general increase in the feedback responses throughout the movement compared with the baseline condition, whereas in the early velocity peak condition we predict a general decrease in these feedback responses compared with the baseline condition. Thus, we show that within the OFC the time-to-target is critical for the regulation of feedback responses, and when we take this into account, we are able to replicate the feedback intensity modulation of our participants.

**Figure 8.**
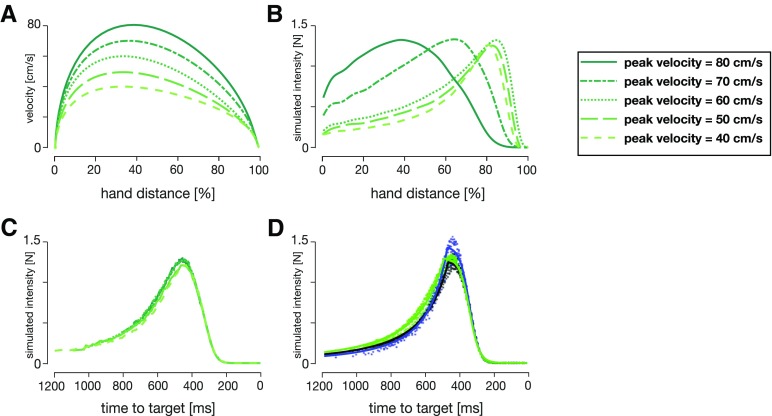
OFC simulations of (***A***) velocity profiles and (***B***) simulated feedback intensity profiles for different desired peak velocities (in order from light to dark line colors: 40, 50, 60, 70, and 80 cm/s). ***C***, Simulated feedback intensities of (***B***) re-mapped as a function of time-to-target at the time of perturbation. ***D***, Simulated feedback intensities vs time-to-target for the three kinematic conditions over the five peak velocities simulated by OFC (colored dots). Solid lines represent the tuning curves (Eq. 7) fit to the data. Both the tuning curves and the simulated feedback intensity profiles are similar across a variety of different kinematics when expressed as a function of time-to-target.

While in our experiment we manipulated the time-to-target through skewing the velocity profiles, time-to-target is naturally modified through changing the peak velocity. Therefore, we can further analyze the effect of the time-to-target by calculating the feedback intensities for movements with different peak velocities (Fig. 8*A*). The simulated feedback intensities vary widely across peak velocities, with a shift of peak feedback intensities toward the earlier locations for faster movements (Fig. 8*B*). However, when these distinct simulated feedback intensity profiles are re-mapped as a function of time-to-target, the simulated feedback intensities follow a consistent, albeit non-monotonic, relationship (Fig. 8*C*). This relationship is also consistent over a range of peak velocities across all three kinematic conditions and is well described by a combination of a square-hyperbolic and logistic function (Fig. 8*D*). The squared-hyperbolic arises from the physics of the system: the lateral force necessary to bring a point mass to a target is proportional to $1/t^{2}$ (Materials and Methods; Eq. 9). The logistic function simply provides a good fit to the data. Overall, our models show that the feedback intensity profiles under OFC are independent of the peak velocity or movement duration. Instead, our simulations suggest that time-to-target is a key variable in regulating visuomotor feedback responses.

**Figure 9.**
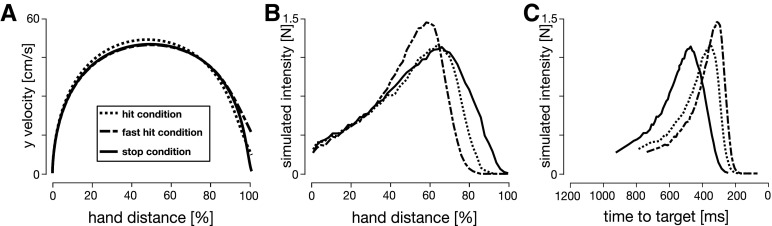
Comparisons between hit and stop instructions. ***A***, Velocity profiles for the stop, hit and fast-hit conditions. ***B***, Simulated feedback intensity profiles as a function of hand position. ***C***, Simulated feedback intensities of (***B***) re-mapped as a function of time-to-target at the time of target perturbation.

It has been shown that the optimal controller gains (Liu and Todorov, 2007), as well as the visuomotor feedback intensities (Knill et al., 2011; de Brouwer et al., 2017) are influenced by task definition (e.g., instruction to hit the target or stop at the target). Here, we simulated the hit, fast hit and stop instructions for our classical model to test how it influenced the relation between simulated feedback intensity and time-to-target. Our previous simulations represent the stop instruction. We modified the *ω_v_* and *ω_f_* to simulate the baseline equivalent of hit and fast hit instructions. Specifically, we set $\omega_{v,hit}=\omega_{v}/4=0.05,\omega_{f,hit}=\omega_{f}/4=0.005$ for hit instruction, and $\omega_{v,fasthit}=\omega_{v}/10=0.02,\;\omega_{f,fasthit}=\omega_{f}/10=0.002$ for fast hit instruction. As changing the terminal costs also results in a change in peak velocity, we further reduced the desired movement times to N = 800 ms for the hit instruction and N = 750 ms for fast hit instruction, such that all three peak velocities match (Fig. 9*A*). According to our simulations, such modification of task demands produced different simulated feedback intensity profiles (Fig. 9*B*). However, the intensity relationship with time-to-target maintained the same structural profile independent of the task demand (Fig. 9*C*). Specifically, both the squared-hyperbolic and logistic segments of the control are still present, although we observe the shift in the temporal location of the crossover point. While each task requires a different pattern of feedback gains (and will therefore produce different responses), variations of the kinematic requirements within a task do not change these gains and therefore do not require recalculation.

### Receding horizon and infinite horizon control

**Figure 10.**
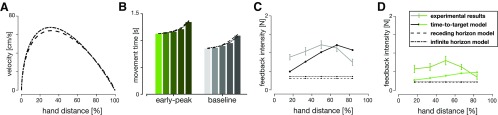
Receding horizon and infinite horizon model simulations. ***A***, Simulated velocity profiles of receding horizon (dashed) and infinite horizon (dot-dashed) models. Both models naturally produce positively skewed velocity profiles, more closely resembling early-peak velocity, rather than the baseline condition. ***B***, Mean experimental movement durations (bar chart) compared with the receding and infinite horizon model predictions. Both models accurately simulate the variations in the reach durations with perturbation location. Baseline (***C***) and early-peak velocity condition (***D***) simulations for receding horizon, infinite horizon and time-to-target (dot-solid lines) models, compared with the experimental data. Only the time-to-target model predicts different visuomotor feedback response intensities for different perturbation onset locations, while receding and infinite horizon models predict constant intensities. Note that models were not fit to match the intensities, only to qualitatively demonstrate the behavior.

A limitation of the finite-horizon implementation used in classical and time-to-target models is that the variable movement duration (Fig. 6) is the model input rather than output. Therefore, in addition to finite-horizon models we also modeled our task in receding and infinite horizon for a single movement condition. Specifically, for the infinite horizon model both state-dependent and regulator costs were kept constant throughout the simulated movement. For the receding horizon model, the regulator cost was kept constant, while the state-dependent cost was zero for all but last “foreseeable” state. Such models were expected to simulate the baseline experimental condition, however the resultant velocity profile better resembled the early-peak condition (Fig. 10*A*). As a result, we compared these simulations with both baseline and early-peak velocity condition data and with the time-to-target model simulations (Fig. 10*B–D*).

Both receding horizon and infinite horizon LQG models were able to successfully capture the nonlinear change in trial durations for different perturbation onsets (Fig. 10*B*) matching the experimental results. In addition, these models also predicted variable times-to-target for the five perturbation onset locations: 700, 660, 620, 600, and 580 ms for the infinite horizon and 690, 640, 610, 610, and 600 ms for the receding horizon. However, neither model showed variation of the simulated feedback intensities for different perturbation onset locations (Fig. 10*C*,*D*), a result that was present in the experimental data and captured by our time-to-target model. Instead both models predicted constant feedback intensities for all perturbations locations. Therefore, neither the receding nor the infinite horizon models are able to explain our experimental results. While both of the approaches can accurately capture the variability in movement duration, only the time-to-target model well describes the behavioral variation in visuomotor feedback responses.

### Quantitative model comparison

**Figure 11.**
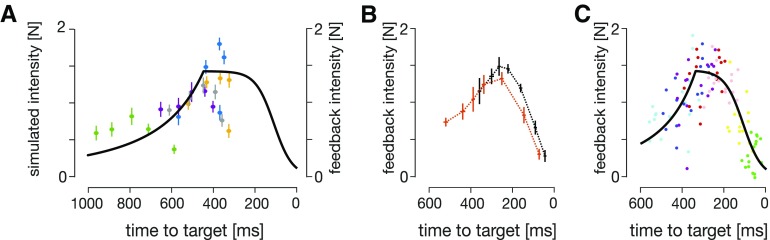
Validation of the time-to-target model. ***A***, Experimental visuomotor feedback intensities for all five experimental conditions (scatter plot) overlaid with the time-to-target tuning curve. The data and the tuning curve show similar qualitative features. Error bars represent 1 SEM. Marker colors indicate five experimental conditions as described in Figure 2*B*. ***B***, Experimental data of the visuomotor feedback intensities of Dimitriou et al. (2013), mapped against the time-to-target. Black and orange traces represent mean participant data for 17.5 and 25 cm movement conditions, respectively. ***C***, A scatter plot of individual subjects’ data from ***B***, overlaid by the time-to-target tuning curve. Both, 17.5 and 25 cm movement conditions are combined to a single representation. Different colors represent different perturbation onset distances as in Dimitriou et al. (2013).

Qualitatively, our results suggest that the time-to-target is an important variable when correcting for visual perturbations in a visuomotor task. In order to supplement these findings quantitatively, we also evaluated model fits between the data and the models using BIC and calculating the SSR for each of the models. We compared the OFC-based models with respect to the classical, finite horizon OFC model as our baseline model. Consistent with our qualitative estimations, the time-to-target OFC model performed the best of all OFC-based models (Δ *BIC_ttt_* = 38.2). In addition, both receding-horizon and infinite-horizon models provided bad fits to the data ($\Delta BIC_{rec}=-23.4,\Delta BIC_{inf}=-18.4$). We also compared, using SSR, the fit of our OFC models with the simpler time-to-target tuning curve (Eq. 7; Fig. 11*A*). While the tuning curve is only a simple approximation to the time-to-target adjusted OFC feedback predictions, it is still able to explain a similar amount of variance in the data (*R*^2^ = 0.33; $SSR_{tuningcurve}=28.5,SSR_{classical}=33.3,SSR_{ttt}=24.9,SSR_{inf}=SSR_{rec}=38.9$). Overall, both the BIC and SSR comparison confirms that the time-to-target OFC model best explains the feedback modulation during human reaching and suggests that time-to-target is a critical variable in online control.

Overall, our simulations suggest that, independent of movement kinematics (different temporal position, velocity, and acceleration profiles), the visuomotor feedback intensities follow the same profile with respect to the time-to-target. We further verified how our time-to-target prediction matches our actual experimental results by plotting participants’ visuomotor feedback intensities against the average time-to-target for the respective perturbation locations and movement conditions (Fig. 11*A*). Specifically, the intensities monotonically increase with decreasing time-to-target until the peak (following the squared-hyperbolic function) and then reduce (the logistic function range).

### Validation of the time-to-target model

We also compared the prediction of the time-to-target model to independent results from an external data set (Dimitriou et al., 2013). In the article, the authors could not rigorously encapsulate both conditions within a simple relationship to movement distance, movement fraction or movement velocity. We plotted visuomotor feedback intensities against time-to-target for two experimental conditions: goal directed reach of 17.5 cm and of 25 cm (Fig. 11*B*,*C*). Two observations can be made from these results. First, the time-to-target model prediction and the experimental data follow the same qualitative features, independent of the target distance (experimental condition). Second, the feedback intensities for both conditions are well explained by a single relationship with time-to-target (Fig. 11*C*; *R*^2^ = 0.56, *SSR_tuning__curve_* = 2.3). Thus, the validation against an external dataset supports our results that visuomotor feedback intensities vary with the time-to-target.

Finally, we evaluated our optimal control models (the classical and the time-to-target) on this dataset. Similar to our original fitting, we fit both of our OFC models to match the kinematics of the human participants, and then simulated the virtual experiment to extract simulated feedback intensities for the models. We found that model parameters $\vec{\omega_{p}}=[0.5,1],\;\omega_{v}=0.03$, and *ω_f_* = 0.03 and R(*t*)* *=* *0.00000235 provide the best fit of kinematics between OFC models and data. As with our data, the time-to-target OFC model provided a better fit of the simulated feedback gains than the classical OFC model (Δ*BIC *=* *19.2, *SSR_ttt_* = 8.6, *SSR_classical_* = 10.7). Together, both our data and Dimitriou et al. (2013) data strongly support our time-to-target model.

## Discussion

Here, we examined how movement kinematics regulate visuomotor feedback responses. Participants extended their movement duration after perturbations to successfully reach the target. In addition, visuomotor feedback responses were modulated when the hand followed different kinematics, but not when the cursor followed different kinematics. In order to better understand this modulation we built four normative models using OFC: a classical finite-horizon OFC (Liu and Todorov, 2007), a finite-horizon time-to-target adjusted OFC, a receding-horizon OFC (Guigon et al., 2019), and an infinite-horizon OFC (Qian et al., 2013). While the classical, receding and infinite horizon models failed to predict the experimental visuomotor feedback response intensities, the time-to-target model qualitatively replicated the visuomotor feedback intensity profile of our participants. Overall, OFC models suggested that feedback intensities for each perturbation location depended on the time-to-target (i.e., time between perturbation onset and target interception) rather than distance or velocity. Moreover, this explains why any mismatch between visual and haptic kinematics had no effect on the feedback intensities, as these manipulations did not affect the time-to-target. Simulated feedback intensities under all movements followed the same profile with respect to time-to-target, suggesting a critical role in the regulation of visuomotor feedback responses.

Experimentally, our participants exhibited a temporal evolution of visuomotor feedback intensities for each condition, confirming the findings of Dimitriou et al. (2013). In addition, we also showed the regulation of visuomotor feedback responses across conditions, allowing us to investigate the underlying mechanism of this temporal evolution. Specifically, our experimental results demonstrated strong regulation of visuomotor feedback intensity profiles with different hand kinematics, but not with different cursor kinematics (Fig. 3*C*). Compared with the baseline condition, in the matched-cursor early-peak velocity condition participants produced longer times-to-target at each perturbation location (Fig. 6*B*), resulting in weaker feedback responses based on the relationship between time-to-target and visuomotor feedback intensities (Fig. 11*A*). The opposite is true for the matched-cursor late-peak velocity condition. As the two matched-hand conditions produced similar times-to-target as the baseline due to similar hand kinematics, we did not observe a different regulation in feedback responses. Therefore, the condition-dependent visuomotor feedback response modulation exhibited by our participants meshes nicely with a control policy whereby the time-to-target regulates the feedback responses.

It has long been suggested that we select movements that minimize the noise or endpoint variability (Harris and Wolpert, 1998). Within the framework of optimal control, this idea has been expanded to the corrective movements, that is, optimality in reaching movements is achieved in part by minimizing the noise during any corrective response (Todorov and Jordan, 2002). As motor noise scales proportionally to muscle activation (Jones et al., 2002; Hamilton et al., 2004), one way of minimizing such noise is reducing the peak levels of muscle activation during the correction. Mathematically, the optimal solution to correct any perturbation approximates a constant activation, resulting in a constant force for the whole duration between perturbation onset and target interception. Such a solution assumes that the brain is capable of estimating the remaining duration of the movement (McIntyre et al., 2001; Benguigui et al., 2003; Zago et al., 2004) and that the force follows the squared-hyperbolic relationship to this duration (Eq. 9). The parallel can be drawn here between our results and the results of Oostwoud Wijdenes et al. (2011), where the authors showed a similar temporal evolution of peak acceleration against the time-to-target in a single forward velocity condition. Our results further show that time-to-target strongly modulates visuomotor feedback responses across a range of different kinematics, consistent with the idea that human participants aim to behave optimally. More specifically, we suggest that, among different optimality variables, the temporal evolution of visuomotor feedback response intensities serves to reduce effects of system noise.

Finite-horizon OFC predicts a time beyond which feedback responses are suppressed. Beyond this critical time, a logistic function well describes the relation between time-to-target and feedback responses, with response intensities reducing as the time-to-target decreases. The controller gains at this stage are the most sensitive to acceleration, suggesting a more “behavioral” outcome, the controller is trying to stop, rather than correct errors. The neural recordings in rhesus macaque monkeys’ supplementary motor area and M1 (Russo et al., 2019) show that supplementary motor area can signal movement termination as far as 500 ms before the end of the movement. This further suggests that there may be multiple stages within a movement, where our control system might “care” more about error correction in one or movement termination in another. On the other hand, the suppression of responses close to the target leads to undershooting the target. Our participants, however, had to bring the cursor to the target to advance to the next trial. As a result, they extended the movement durations postperturbation to return to the squared-hyperbolic range of control. The control performance of such behavior is well accounted for by our time-to-target model. Moreover, our time-to-target model also well explained the modulation of visuomotor feedback intensities from an external data set (Dimitriou et al., 2013). However, an important distinction from our study is that in Dimitriou et al. (2013), the suppression of feedback responses toward the end of movements would not interfere with reaching the target as perturbation trials were always in a mechanical channel so that no corrections were required. As a result, the times-to-target were shorter and the data clearly exhibits both logistic and squared-hyperbolic segments of the control.

All of the variations of optimal control models are formulated as two controllers in *x*- and *y*-axes (coupled through control-dependent noise), with no modeling of the musculoskeletal dynamics. However, the experiments were performed using multijoint reaching movements of the arm. One possibility is that the presence of these musculoskeletal dynamics in the human participants could explain the differences in the feedback intensities, as the matched cursor conditions required different hand accelerations. One might therefore imagine that the condition with the fastest initial movements (early-peak velocity) requires the largest initial forces and could therefore produce larger initial feedback intensities as a default. However, several studies have shown that there is no scaling of visuomotor feedback gains with background loads or muscle activity (Franklin et al., 2012, 2017). More critically, this condition actually shows the lowest feedback gains early in the movement, whereas the condition with the slowest initial acceleration produces the highest feedback gains. Therefore, we suggest that the neuromuscular dynamics cannot explain the modulation of these feedback responses.

Both of the matched cursor conditions (early and late peak) require a change in the physical kinematics away from the naturally occurring bell-shaped velocity profile. One possibility is that this manipulation could have driven the changes in feedback intensity. We argue against this possibility for two reasons. First, all participants were able to fairly quickly learn this pattern of movement with training before the testing of the feedback intensities. Second, if this manipulation away from the naturally occurring bell-shaped profile affects these feedback intensities, we would expect the feedback gains in these two conditions to either both increase or both decrease. Instead we find that the feedback intensity profile for each condition changes in a manner that is explained by the change in the time-to-target.

A limitation of our time-to-target model is that it takes time-to-target as an input to generate feedback intensity predictions, rather than obtain the time-to-target as a model output. As a result, our time-to-target model does not describe exactly how the change in movement geometry after the perturbation influences this time-to-target, which in turn regulates the visuomotor feedback responses. On the other hand, both receding and infinite horizon models did predict the movement duration change after perturbations very well, but could not at all describe the changes in visuomotor response intensity. However, utility of movement has recently been used within optimal control to characterize reaching movements (Rigoux and Guigon, 2012; Shadmehr et al., 2016) in which optimal movement time falls out automatically from a trade-off between reward and effort. With respect to our models, this adds additional complexities to capturing the different movement conditions. Future approaches could attempt to model these results within the utility of movement framework.

In addition, our time-to-target model does not directly show the causality of the time-to-target as a control variable for the visuomotor feedback intensities. Particularly, the time-to-target relation to feedback intensity could be a by-product of a more sophisticated control scheme. Additional arguments for the time-to-target control scheme could be two-fold. First, there is evidence that humans are well capable of estimating the time-to-target of a moving stimulus, even if it is accelerating (McIntyre et al., 2001; Benguigui et al., 2003; Zago et al., 2004), indicating that time-to-target is at least an available input for such a controller. Second, while we have tested finite-horizon OFC and two other (receding and infinite horizon) OFCs, only the finite horizon controllers had any effect on the variation of simulated feedback intensities. Importantly, neither the receding nor infinite horizon models use time-to-target as an input to the controller. We posit that this time-to-target control input is the one key difference between the finite and non-finite models and is therefore the simplest explanation for our results.

Our results show that models incorporating time-to-target (the time-to-target OFC and the simple time-to-target tuning curve) better describe our experimental data and those of Dimitriou et al. (2013) than do other optimal control models. Specifically, the relative scaling of the conditions is explained using the time-to-target tuning curve (Fig. 11*A*): the times-to-target are longer for the early-peak velocity condition compared with the baseline, and therefore fall in the lower intensity range (and vice versa for the late-peak condition). However, there are still some qualitative differences between the experimental and model predictions. That is, our experimental results exhibited an inverted U-shape for the feedback intensity profiles, whereas the model predicts only a slow increase in some conditions (Fig. 8*C*, green curve). Our time-to-target model suggests that this inverted U-shape is not characteristic of the feedback intensity profile, but is simply an outcome of the experimental design (particularly the reaching duration). Indeed, the time-to-target model makes specific predictions about the feedback intensities for much faster movements, which should not show an inverted U-shape but instead decrease throughout the movement. Our model, therefore, makes strong predictions that can be tested in future studies.

Rapid feedback responses scale with the temporal urgency to correct for mechanical perturbations (Crevecoeur et al., 2013). Here, we have shown that visuomotor feedback responses also follow a similar regulation, suggesting that these two systems share the same underlying control policy. Our work further extends this finding of Crevecoeur et al. (2013) by not just showing that temporal urgency affects feedback responses, but explaining the manner in which these responses are regulated with respect to urgency. That is, here, we have shown that for visual perturbations the feedback intensities scale with a squared-hyperbolic of the time-to-target, which is a direct measure of urgency. Moreover, the feedback intensities were rapidly adjusted due to the change in urgency as the task changed. Specifically, when the cursor jumps close to the target, the expected time-to-target is prolonged, and therefore the optimal visuomotor feedback response needs to be adjusted appropriately to this increase in time. Our results show that participants produce a visuomotor response consistent with the actual, postperturbation, time-to-target, as opposed to the expected time-to-target before the perturbation. Therefore, our results not only suggest that similar computations might occur for both stretch and visuomotor feedback response regulation, but also that this regulation originates from task-related OFC.

Our proposed time-to-target model is not meant to contradict the conventional OFC models, but rather show that the OFC could be approximated by a simple time-to-target control. Our work has shown that simulated feedback intensities from OFC exhibit the same underlying pattern as a function of time-to-target over a wide range of movement kinematics, matching well the feedback intensities of our human participants (Fig. 7). As expected, changes in the task goals (e.g., hit vs stop) changed the relation between feedback responses and time-to-target. However, the qualitative features, the squared-hyperbolic and logistic function, remained consistent across these tasks. These results suggest that, for a specific task and known dynamics, we do not need to recalculate the feedback gains before each movement, but instead can access the appropriate pattern as a function of the estimated time-to-target in each movement. Therefore, gain computation in reaching movements may not be a computationally expensive process, but instead could be part of an evolutionary control strategy that allows for rapid estimation of the appropriate feedback gains. Moreover, the fact that both stretch reflex and visuomotor feedback systems exhibit similar control policies despite different sensory inputs, perhaps only sharing the final output pathway, suggests that this simple feedback pathway may be an evolutionary old system. Indeed, several studies have suggested that visuomotor feedback is controlled via a pathway through the colliculus (Corneil et al., 2004; Reynolds and Day, 2012; Gu et al., 2019). Furthermore, it has been suggested that visuomotor feedback responses involve two different phases that are behaviorally different Cross et al. (2019). This might reflect two different pathways, the early through the colliculus and the later through cortex. The nature of our analysis only focuses on the earlier of the two phases, which shows limited sensitivity to environment, but is still sensitive to goal redundancy. We suggest that this limited sensitivity could be the outcome of the time-to-target model in action, providing simplified, yet still flexible control in the early phase of the visuomotor response. Such a system would then only need to be adapted as the dynamics or overall task goals change, allowing for fine tuning of the feedback gains according to changes in the environment (Franklin et al., 2017).

Our results have shown the connection between the visuomotor feedback response regulation and the time left to complete the movement. Specifically, in our human participants we recorded the increase in the time-to-target after the perturbation onset, which consequently increased the movement durations (Fig. 6). This increase was also longer for later perturbations, consistent with previous studies (Liu and Todorov, 2007). According to our normative time-to-target OFC model, the time-to-target alone is enough to successfully regulate visuomotor feedback responses as observed in humans. This result was independent of the physical kinematics of the movement or the onset times of the perturbations. This suggests that there is no recalculation of a control scheme for the rest of the movement after the perturbation, but rather a shift to a different state within the same control scheme. Such findings are consistent with the idea that visuomotor feedback gains are precomputed before the movement, allowing for faster than voluntary reaction times (Franklin, 2016). Moreover, through our results, we gain a deeper insight into how OFC governs these feedback gains, through a straightforward relationship to the estimated time-to-target, based on physics.

## Synthesis

Reviewing Editor: Tatiana Pasternak, University of Rochester Decisions are customarily a result of the Reviewing Editor and the peer reviewers coming together and discussing their recommendations until a consensus is reached. When revisions are invited, a fact-based synthesis statement explaining their decision and outlining what is needed to prepare a revision will be listed below. The following reviewer(s) agreed to reveal their identity: Adam Rouse.

The revised manuscript addressed adequately the remaining reservations of the two reviewers.The paper is now ready for publication in eNeuro.

Please address one minor software comment from Reviewer 2 that should be addressed: “I would recommend that the authors include the relative path of the included data files. For example:

load(‘..\utils\figure_data\Fig4.mat’)

rather than load(‘Fig4.mat’)”.

----------

REVIEWER 1

ADVANCES THE FIELD

I appreciate the authors time addressing the previous comments and adding sufficient clarifications and statistical analyses to the manuscript. Clarifying specific sections also helped to clarify my misunderstanding about what exactly their OFC models were able to predict. I think this is a nice manuscript comparing the classical OFC with their time-to-target OFC and demonstrating that incorporating specific features of the task allows for more accurate represent dynamical changes of the reaching kinematics. The changes the authors have made make it clear why this is important when comparing to classical models.

COMMENTS TO THE AUTHORS

I appreciate the effort the authors have made to clarify misunderstandings of what their model is capable of predicting (i.e., can’t predict kinematics). Their clarifying statements and additional use of the BIC to compare models is appreciated. All of my comments/concerns have been adequately addressed.

REVIEWER 2

ADVANCES THE FIELD

I believe the experiment demonstrates a novel way to vary time-to-target for a visuomotor task and demonstrates differences in the feedback response to visual perturbations.

SOFTWARE COMMENTS

I was able to locate the matlab scripts and did a spot test and was able to get it to work. I would recommend that the authors include the relative path of the included data files. For example:

load(‘..\utils\figure_data\Fig4.mat’)

rather than load(‘Fig4.mat’)

COMMENTS TO THE AUTHORS

The authors have addressed all of my previous concerns and I have no further concerns.

## Author Response

Synthesis Statement for Author (Required):

The paper was carefully reviewed by two reviewers who raised a number of serious reservations which must be addressed for the manuscript to be considered for publication in eNeuro.

The most important reservations include:

1. the absence of strong evidence that the time-to-target is the main variable that is being modeled and the lack of discussion of alternative variables governing the task and the absence of consideration of alternative models.

2. the lack of statistical evidence that the OFC model with time-to-target as a variable is the best model to account for the presented data and the lack of direct and quantitative comparison of this model to the other OFC models.

3. Both reviewers made a number of more detailed comments and suggestions that I recommend you address in the revision, if you decide to resubmit.

Response:

We compare traditional optimal feedback control, receding horizon optimal control, infinite horizon optimal control and our extension of optimal feedback control where we consider updates of the time-to-target. We now add supporting statistics for the comparison of all of these models, rather than only rely on the visual comparisons. Importantly our model is also able to better represent the results of previous (independent) experimental data than other models. We have made changes to the manuscript and addressed each of the reviewer’s comments in detail.

REVIEWER 1

Advance the field

In this manuscript, entitled “Time-to-target simplifies optimal control of visuomotor feedback responses” the authors applied different optimal feedback models to characterize how reaching kinematics map to cursor position on a screen. For catch trials, the trajectory of the cursor was perturbed at different distances from the target to observe how the participants reach kinetics adapted to correct for the perturbations. OFC strategies were used to model the dynamics of this visuo-motor task, in which incorporating information about the time-to-target seemed to better represent the reach kinematics and perturbation responses. Detailed simulations were conducted, however none of the models actually characterized the experimental findings. Comparisons of the models were mainly qualitative and the argument that including time-to-target did not seem very strong without more quantitative metrics/evidence.

Response:

We have now added quantitative metrics. We think one major mis-understanding of the reviewer is stated above. We never use the models to predict the reach kinematics. The only comparison between the models and the experimental results is done for the feedback gains. Indeed, we cannot compare the kinematic predictions as we require the kinematics as an input (in terms of extension of the movement duration) to the time-to-target optimal control model. We believe that it is this critical issue that has led to several misunderstandings below. We have added a sentence into the methods of the paper to avoid this misunderstanding for other readers.

Reviewer’s comment:

Statistics

There are several results in which the data are qualitative described or compared but could benefit from a statistical comparison. For example, Figure 4C, D show different regression slopes for the different reach conditions however no statistical test appeared to be performed (unless I missed it).

Response:

In this figure we provide 95% confidence intervals of the mean for each of the slopes. The 95% confidence interval has statistical meaning, such that if the intervals do not overlap, the slopes are significantly different at p<0.05.

Reviewer’s comment:

In this manuscript, entitled “Time-to-target simplifies optimal control of visuomotor feedback responses” the authors applied different optimal feedback models to characterize how reaching kinematics map to cursor position on a screen. For catch trials, the trajectory of the cursor was perturbed at different distances from the target to observe how the participants reach kinetics adapted to correct for the perturbations. OFC strategies were used to model the dynamics of this visuo-motor task, in which incorporating information about the time-to-target seemed to better represent the reach kinematics and perturbation responses. Detailed simulations were conducted, however none of the models actually characterized the experimental findings. Comparisons of the models were mainly qualitative and the argument that including time-to-target did not seem very strong without more quantitative metrics/evidence. Had the models quantitatively matched the experimental findings, then this would have been more convincing that mainly including time-to-target is the main source of modulation when perturbing the visuomotor feedback.

Response:

Our model comparison is generally qualitative. However, it is important to note that this is sufficient for most of the models. Importantly both the receding horizon and infinite horizon models predict no variation at all in the feedback gains, whereas our measurements of feedback gains show otherwise. However, we agree that more quantitative metrics would be useful and have added them to the paper. Specifically, we now show quantitatively, that the time-to-target generally improves the model predictions. The time-to-target OFC model is better capable of capturing the variance in the data then the classical model (lower SSR), and is a better overall fit (lower BIC) than other models. This result is consistent in both our and Dimitriou’s data. Finally, the time-to-target tuning curve is now also evaluated not only qualitatively by its features, but also by calculating SSR (which is comparable with the one for OFC-based models, even though the tuning curve is much simpler).

Reviewer’s comment:

I have the following comments:

- My one concern involves whether the finding that incorporating time-to-target into the model results in a better fit of the data is a result of how the experimental setup was designed. In the introduction, the authors describe how visual or haptic information could be used to control movements. They then focus on visual-motor feedback and apply different velocity curves which will map based on the hand position or the cursor position. However, I’m wondering whether the finding that models including time-to-target is more a result that the main source of variance in the task is time-to-target. The authors acknowledge on lines 341-348 that experimental conditions inherently require changes in time-to-target. The participants are performing several trials at very consistent speeds in order to perform the task correctly, thus averaging over all the trials will appear to be a very consistently timed reach. So it’s good that the model accounting for the time-to-target fits better than not accounting for it, because that appears to be a strong signal in the task structure. However, I’m not sure the result is that surprising.

Response:

Inducing variance of time-to-target was indeed desirable for our experimental design. If all conditions had exactly the same time-to-target, it would not be possible to see any effect of time-to-target. This, therefore, would not allow us to contrast the traditional optimal feedback control model with our time-to-target model as they would both predict exactly the same patterns of feedback gains. However, it is very important to mention that we are not interested in explaining variability in the time-to-target and do not try to do so. Instead both the output of the models and the output that we use to compare models is the strength of the feedback gain, the measurement of which is independent of any change in time-to-target. Importantly, independent of whether we induced time-to-target dependent variability or not, if it is not an important parameter, adding it to the model would not improve it. We have made this point clear in the Methods. Moreover, we provide further support for our model using the data from Dimitriou et al (2013) that does not have any extensions in time after the perturbation. We believe this concern arises from an important misunderstanding as described above. Specifically, we do not use the models to predict changes in kinematics or movement durations as these are inputs to our model.

Reviewer’s comment:

What would have been interesting is if the time-to-target was the largest source of variance when reaching to a target using different sources of feedback (e.g., visual, auditory, somatosensory/haptic). Or if the task was extended to reach to and grasp objects at different locations (or at least multiple targets rather than the one shown here) does OFC still require time-to-target for the “best” representation of modeling the data.

Response:

This is an interesting line of research but it is well outside the results of our current manuscript.

Reviewer’s comment:

- A schematic of the 5 reaching conditions, a timeline of the trial structure, and a picture/drawing of the setup would be helpful to have a better understanding of how everything was conducted. I found myself confused at times because the different trial conditions were better described in certain parts of the text than others. The description of the hand velocity and cursor velocity was slightly confusing at first when I read it in the methods, However, in the figure legend the line “Feedback was provided on the modality (cursor or hand) that matched the baseline” made this clearer which could help clarify the text (lines 114-125).

Response:

We provide the schematic to five different conditions and five different trial types in figures 1 and 2A. We provide further references to the setup (Howard 2009), which includes drawings of the set-up. The same set-up can be found in other papers as well - for example Dimitriou et al., 2013 (J Neuroscience). We also include drawings of the feedback provided on each trial (Figure 1). We state explicitly that each session consisted of 40 similar blocks, where the order of trials was randomized within each of the blocks (revised manuscript lines 200-207). We have gone through the material and made edits where required to ensure that all critical descriptions are included.

Reviewer’s comment:

- Much of the results involve comparing different models, however it did not seem like these models were really quantitatively compared (mainly qualitative descriptions). Couldn’t a goodness of fit metric based on inputting the experimental data and comparing to the actual data be measured for each model to justify that the OFC with time-to-target was “best”? The lack of quantitative comparisons of the models based on the experimental data weakens the arguments here. The time-to-target model had a feedback intensity response that modulated with hand distance, but it didn’t actually match the experimental data. So does that mean it’s the correct model or interpretation of the model? The authors address this in their discussion section, but it severely weakens the arguments set out in the abstract that “time-to-target is a critical control parameter in online feedback control” (line 26) when in fact that hasn’t actually been proven here. In general, more quantitative comparisons of the models would be needed to validate the claims here and more convincingly relate this to the underlying neural control of reaching.

Response:

As we outlined in the previous comment regarding this issue we have now included quantitative statistics to support our claims. These results support the qualitative differences in the models that we previously explained.

Reviewer’s comment:

I have the following other comments:

Abstract

- I’m confused by the statement “only the model with time-to-target as one of the input parameters can successfully replicate the experimental data,” to what extent does it replicate? Identically? Or is this more meaning that the time-to-target models provide the best fit of the experimental data (which is a slightly different statement). (line 25)

Response:

We have revised this statement to: “the model with time-to-target as one of the input parameters best replicates the experimental data.”

Reviewer’s comment:

- Similarly confusing statement, “humans can instantly produce a control signal without any computation allowing rapid response onset and close to optimal control.” What is meant by producing a control signal without any computation? Presumably the nervous system is performing numerous computations which I assume is then resulting in a control signal. Do the authors mean that there is a signal that is a direct readout?

Response:

Yes, we mean precisely this. We mean that the generic computation of control landscape happens before the movement (to build-up the TTT-gain dependence function) and from then on it is a direct readout. We have revised this statement.

Reviewer’s comment:

Also, why would this be restricted to humans (e.g., non-human primates might have a similar readout)? (line 27-28)

Response:

We agree with the reviewer, but we have only studied humans so this is all we can state in our manuscript.

Reviewer’s comment:

Intro

- Is the phrase “visual or haptic kinematics” the interaction between visual and haptic information with the hand kinematics? (line 65) or are you trying to say that the modulation of kinematics (position, velocity, acceleration) might be more dependent on either visual or haptic information about the movement?

Response:

We are saying that there is an integration of multiple sources of information. Here we do not know yet whether visual kinematics or haptic kinematics are more important in contributing to any control gains. We have revised this sentence to make this clearer.

Reviewer’s comment:

- Why is haptic or somatosensory-motor integration introduced when it doesn’t seem to be a focus of the study?

Response:

Our experimental results clearly rejected the effect of visual kinematics (Fig 3A, Fig 4), so we do not include this in further modelling - lines 424-426 of the revised manuscript. We now better explain our motivation of the experimental design in the Methods.

Reviewer’s comment:

Methods:

- what was the graphics card refresh signal rate? Also is this correct or wouldn’t it be dependent on the refresh rate of the computer monitor used (and not necessarily the graphics card itself)? (line 100)

Response:

Yes, our statement was correct. The goal of it is to know exactly where any new visual stimulus will be displayed on the screen and at what exact time. We detect the graphics card refresh signal (not the rate) in order to learn when the new visual stimulus will be redrawn. This approach has been fully verified using optic sensors in several papers (see for example Dimitriou et al., 2013).

Reviewer’s comment:

- How many targets are on display before one of them is selected? Also when was the target indicated? Before, after or at the same time as the go cue? (lines 101-106)

Response:

There is only one target in the whole experiment. The target is indicated before the go beep. The cursor jumps (where applicable) after the participants are already moving at different points in the movement (Fig 3A, lines 148-157 in the original manuscript; lines 175-185 in the revised manuscript).

Reviewer’s comment:

- Could participants enter and exit the target or did they need to acquire the target and hold it continuously for 600 ms? (e.g. is it a cumulative 600 ms or continuous 600 ms)?

Response:

They needed to be in the target continuously for 600 ms. We have clarified this in the paper.

Reviewer’s comment:

- I just wanted to clarify that undershooting or overshooting would be an error? Or only if there was under and overshooting that lasted over 4 seconds that would then result in an error? (if the latter, how would one handle these corrective movements)? (lines 108-110)

Response:

If participants undershoot, they never arrive at the target. 4s after the start of the movement the movement would time-out if the cursor is not at the target and the movement would have to be repeated. Overshooting was recorded even if participants arrived at the target, but if they went beyond the target and came back. In such a case the movement would be considered as complete, but the error message would be displayed and the point would not be given, even if the movement was otherwise perfect. We have clarified this in the paper. Further details are also found in the section titled “Feedback regarding movement kinematics”.

Reviewer’s comment:

- Why was the scaling parameter set to range between 40% and 160% of the hand/cursor velocity? (lines 124-133) Also, I’m confused by 40% of the hand velocity at the beginning of the movement, isn’t that inherently zero since at the beginning of the movement no movement has occurred yet? It might help to define the trial structure and indicate where after the go cue this scaling is occurring

Response:

The scaling parameters were selected so that the two movements (hand and cursor) would start and stop simultaneously at the two same positions (start and target). This could have been achieved with other values too (for example 30% and 170% as well as others), but these different values shift the peak velocity locations between visual and proprioceptive signals. These locations were our design choice (importantly, that allowing for motor noise the three different profiles would be significantly different). Scaling is occurring exactly as described - throughout the entire movement. When the hand is stationary the cursor is also stationary. Any scaling value multiplied by zero (velocity) gives a value that is still zero.

Reviewer’s comment:

- Were the participants instructed that there would be different mappings on each session? If the participants expected each session to occur like the baseline session compared to knowing there would be a change might affect some of the results. Also, what order were the sessions performed for each participant? Was baseline always first? Was it pseudorandom after that or the same order?

Response:

L 204-205: The order of the five different conditions (sessions) was pseudo-randomized and counterbalanced across participants.

Participants were not told about the physical implementation of the mappings, but (A) had constant feedback after every trial of their performance, (B) knew that every session was different from before and (C) were provided training before the main experiment to adjust to new profiles.

We have added further details to the manuscript.

Reviewer’s comment:

- Perturbations: I’m not sure if I understood this correctly. Are the perturbations only visual or is the manipulandum also perturbed?

Response:

Only visual perturbations were applied (lines 175-185, but specifically L177 of the revised manuscript).

Reviewer’s comment:

After the perturbation is the participant required to move in 2 directions to adjust the cursor position back to the straight line? If so, it might be worthwhile analyzing the velocities in 2D and not one as shown in Figures 3-4.

Response:

Our probe trials, as described in lines 175-185, constrain the participant’s hand in a virtual channel, so there is no movement in x-direction. Instead, we measure the corrective force in that direction, which participants applied as if to correct for the perturbations. On the non-probe (maintained perturbation) trials, the x-velocity does not provide us with any new information. Particularly, we know that our x-acceleration will look the same as the x-force in probe trials, and velocity is just the integral of that.

Reviewer’s comment:

Results

- (lines 328-330): if the mapping had to be learned, can you look at the performance of the task over the course of the session to see how the velocities and movements became more/less variable? Also was there any effect of what session occurred before this session?

Response:

Looking at the variability over time would not be helpful for our current claims.

We counterbalanced the order of the sessions to specifically remove any possible effect of the order, because it is not what we look at with this study.

Reviewer’s comment:

- When comparing the slopes in figure 4, was there a statistical test performed here? (lines 334-340)

Response:

As described above, 95% confidence intervals indicate statistical significance.

Reviewer’s comment:

- In addition to looking at the movement times, it might be interesting to look at the movement distances (although this might just be the same thing)

Response:

We did exactly this - Figure 3C and Figure 7.

Reviewer’s comment:

- For Figure 5 results, I’m confused by Figure 5A. The shortest distance increase appears to relate to traces that occur earlier and appear to have a longer trajectory, what is defined as the distance increase? Is it the net distance of the trajectory traveled? And I’m even more confused when the text says that these are corrected angles (lines 341-342). So are these arc lengths in cm? It’s also interesting that these perturbation locations listed do not align with when the trajectories begin to deviate from 0?

Response:

The participants always need to reach the target. Therefore, in all conditions the minimum distance is 25.0 cm (for example when there is no perturbation). We define the increase in distance as any travelled path larger than this minimum distance. If corrections occur earlier, then participants are able to still reach the target with almost no increase in the distance. If the corrections occur later then participants need to produce a corrective movement that is much larger. We have edited the text to make this clearer for the reader.

The corrections will always be later than the perturbation locations because there are delays in the human motor control system. Extensive previous work has shown that there is always a delay between a stimulus (in this case a visual shift) and a motor response (change in path kinematics).

Reviewer’s comment:

- When simulating the data, why did the authors decide to go from 0 to 100% distance when the experimental data did not range from here?

Response:

Optimal control models are simulated along the whole trajectory. This was also critical to show that the feedback intensities are the same across many different kinematics (Figure 8).

Reviewer’s comment:

- “Any extension of the movement distance requires an appropriate increase in movement duration.” (line 345-346), this could also be a result of the experimental paradigm because the participant is constrained to moving at a specific velocity range. Just hypothesizing here, but if the requirement was not to maintain a specific velocity but instead to acquire the target as fast as possible wouldn’t that affect these movement times? There still might be a trend but maybe not as pronounced?

Response:

This would still always be true as participants cannot make instantaneous movements. Therefore, even if participants were already going as quick as possible, had to move further, this would take more time. Please do not forget that the perturbations cannot be predicted as they occur randomly in different directions at different times.

Reviewer’s comment:

Figures:

- Figure 3: for 3B what does this figure look like for forces instead of velocities? I’m surprised that the green trace in Figure 3C already is lower at d=20% since 3B doesn’t show a significant change in distance at that point in the velocity trace? This might be explained when looking at the forces

Response:

While this might seem surprising, it is important to note that both the optimal feedback control model and our time-to-target optimal control model predict this difference early in the movement. The feedback gains (lateral force to a visual perturbation) can be independent of the forces in the movement at any point during the movement. Specifically, the feedback gains are in the lateral direction, where there are little variations in the forces produced by the participants. In the forward direction there will be differences, as different forces are always required in order to produce different kinematics. However, force will be proportional to acceleration which is the derivative of velocity. Therefore, we can directly see that the forward force for the green condition will be higher earlier in the movement and the forward force for the blue condition will be lower earlier in the movement.

Reviewer’s comment:

- Figure 5A: not sure why this plot ranges from -2 to 2 when there appears to be no data <0

Response:

This was done simply to center the data in the figure.

Reviewer’s comment:

- Figure 7: why are there many fewer time steps in the simulations for B and C?

Response:

As described in the paper, the time-to-target model needs values of any extension in movement duration produced by any perturbation to calculate a different value. We only have values for this extension in movement time for the 5 selected points in which we conducted the experiment. We have clarified this point both in the Methods and in the figure capture.

Reviewer’s comment:

- Figure 11: A) How well do these data actually fit the curve?

Response:

Our original Figure 11A was not actually fit to the data and its purpose was to demonstrate similar qualitative features between data and model. During this revision, as we added additional quantitative analysis, we now fit this curve to the data and its fit is described in the Results. Specifically, the tuning curve demonstrated in Fig 11A accounts for similar amount of variance as the time-to-target OFC model, and more than the classical OFC. Further, we now also provide a similar fit to the data of Dimitriou et al. 2013 in Fig 11C.

Reviewer’s comment:

Overall, the paper is an interesting application of OFC techniques of reaching and perturbations. The authors did a good job of using several different models to demonstrate that theirs appears to match the experimental results. However, I’m just not sure how well this will translate to more complex sensorimotor integration studies of reaching and grasping in which a multitude of sensory information are used to perform more advanced tasks beyond reach and stop at a target.

REVIEWER 2

Advances in the field

I believe the experiment demonstrates a novel way to vary time-to-target for a visuomotor task and demonstrates differences in the feedback response to visual perturbations. I agree with the data not fitting well with a classical OFC model with only distance and velocity state variables and being generally improved with time-to-target. Although I have some concerns about the evidence that the time-to-target model matches the data that well (see Comments to Authors)

This manuscript presents results from a reaching experiment with visuomotor perturbations in which both hand and cursor kinematics were varied relative to baseline bell-shaped reaches. The time-to-target was varied by requiring subjects to move with different hand velocities based on different experimental transformations between the cursor and hand position. The feedback intensity to visual perturbations were observed to vary depending on whether the physical hand was required to have a peak velocity early (less intensity) or late (greater intensity) compared to a standard bellshaped velocity at baseline. Warping the cursor velocity in the forward direction with the no change in hand velocity from baseline had no effect. The authors argue that this response is best accounted for by including a time-to-target state variable in the feedback control model.

Overall, I agree that the presented results show clear and significant differences between the different hand velocity conditions and no change for the different cursor conditions. I also agree with the assessment that the time-to-target model more closely matches the data than other models. There are however some qualitative difference between the time-to-target model and the actual data.

Response:

We thank the reviewer for the positive comments about our manuscript.

Reviewer’s comment:

My concerns:

1) My main concern is the potential interaction between the experimentally manipulated forward velocity of the hand and the lateral feedback corrective intensity. It seems to me that in addition to manipulating the time to target, this manipulation could also influence the musculoskeletal dynamics when requiring the forward velocity of the arm to be different. Although modeled as two independent x and y components, the various complex muscle relationships related to complex force-length, force-velocity, stiffness, and co-contraction as the forward velocity and whether the arm is accelerating and deceleration are different at the time of perturbation for the different conditions.

Response:

Although not critical, it is important to mention that the two separate x-y components are not totally independent as there is a coupling in the form of a control-dependent noise (similar to Liu and Todorov, 2007). We agree that our model does not contain any assumptions about musculoskeletal dynamics and their effects on our results. However, in regards to the reviewer’s concerns regarding the possible effect of the manipulated forward velocity of the hand on the lateral feedback intensity, we can completely rule out this as explaining our results. This is because the condition which has the fastest initial velocity (matched cursor early peak velocity: green), which must be produced by the largest muscle activation and therefore would be expected to produce the largest feedback gains, actually shows the lowest feedback gains early in the movement. Instead the slowest initial movement (matched cursor late-peak velocity condition: blue), requiring the lowest forces, exhibits the largest feedback gains even early in the movement. We have added a discussion of this issue in a paragraph discussing some possible limitations to the current version of the manuscript. Specifically we state:

All of the variations of optimal control models are formulated as two controllers in x- and y- axes (coupled through control-dependent noise), with no modelling of the musculoskeletal dynamics. However the experiments were performed using multijoint reaching movements of the arm. One possibility is that the presence of these musculoskeletal dynamics in the human participants could explain the differences in the feedback intensities, as the matched cursor conditions required different hand accelerations. One might therefore imagine that the condition with the fastest initial movements (early-peak velocity) requires the largest initial forces and could therefore produce larger initial feedback intensities as a default. However, several studies have shown that there is no scaling of visuomotor feedback gains with background loads or muscle activity (Franklin 2012, 2017). More critically, this condition actually shows the lowest feedback gains early in the movement, whereas the condition with the slowest initial acceleration produces the highest feedback gains. Therefore we suggest that the neuromuscular dynamics can not explain the modulation of these feedback responses.

Reviewer’s comment:

Along similar lines, the variations in hand velocity move away from an optimal control strategy of a bell-shaped velocity profile that minimizes the maximal force required and thus require the subjects to perform the task in an unusual domain from the start before the perturbation.

I don’t think these concerns necessarily rise to the level of preventing the results and time-to-target modeling being interesting findings, but these various concerns should be addressed by the authors.

Response:

We have addressed this point in the discussion. We now state:

Both of the matched cursor conditions (early and late peak) require a change in the physical kinematics away from the naturally occurring bell-shaped velocity profile. One possibility is that this manipulation could have driven the changes in feedback intensity. We argue against this possibility for two reasons. First, all participants were able to fairly quickly learn this pattern of movement with training prior to the testing of the feedback intensities. Secondly, if this manipulation away from the naturally occurring bell-shaped profile affects these feedback intensities, we would expect the feedback gains in these two conditions to either both increase or both decrease. Instead we find that the feedback intensity profile for each condition changes in a manner that is explained by the change in the time-to-target.

Reviewer’s comment:

2) The main qualitative difference I see is that that the feedback intensity is upside U-shaped for all 3 hand velocity conditions as a function of hand distance (Fig 7D) while the model would be mostly increasing especially for the baseline and early-peak conditions (Fig 7C). The differences between the 3 hand velocity conditions appear to be mostly a simple scaling of one another. I wonder if the authors could provide any further comments.

Response:

We have added a new paragraph in the discussion to address this issue. Specifically we state:

Our results show that models incorporating time-to-target (the time-to-target OFC and the simple time-to-target tuning curve) better describe our experimental data and those of Dimitriou et al. 2013 than do other optimal control models. Specifically, the relative scaling of the conditions is explained using the time-to-target tuning curve (Figure 11A): the times-to-target are longer for the early-peak velocity condition compared to the baseline, and therefore fall in the lower intensity range (and vice-versa for the late-peak condition). However, there are still some qualitative differences between the experimental and model predictions. That is, our experimental results exhibited an inverted U-shape for the feedback intensity profiles, whereas the model predicts only a slow increase in some conditions (Figure 7C, green curve). Our time-to-target model suggests that this inverted U-shape is not characteristic of the feedback intensity profile, but is simply an outcome of the experimental design (particularly the reaching duration). Indeed, the time-to-target model makes specific predictions about the feedback intensities for much faster movements, which should not show an inverted U-shape but instead decrease throughout the movement. Our model, therefore, makes strong predictions that can be tested in future studies.

Reviewer’s comment:

3) I also believe better descriptions of the rationale for selecting the given experimental paradigm to vary trial-to-target and a better explanation of why the different conditions creates the desired experimental variables could strengthen the Methods. The methods currently provide a description of the how the experiment was implemented but it would be useful if the methods could be better linked to the end of the introduction for why these conditions are a good choice. In my opinion it would also be better if it focused more on what the subject was being trained to do, possibly including the start of the Results or referencing Figure 2 more in the Methods.

Response:

We have revised the Methods and now added the paragraph below to explain the choice of conditions:

Introducing the differences in velocity profiles across five experimental conditions allows us to look at the effect of the time-to-target separately from the kinematics of physical movement. First, as the visual perturbations always occurred at the five preset hand positions, our design allows us to maintain the effect of the distance to the target and distance in the movement constant across all five conditions. Second, two perturbation locations (one-third and two-thirds of movement distance) were chosen so that velocities at those locations matched across multiple conditions (early-peak condition velocity equals baseline at one-third, and late-peak velocity equals baseline at two-thirds), allowing for matching velocity contributions across conditions as well. However, across the three different physical kinematics the time-to-target is varied, allowing us to investigate whether time-to-target has an effect. Finally, the relative scaling between cursor velocity and hand velocity in the forward direction also separates the relative contributions of these two inputs, allowing us to examine the relative contributions of visual and physical kinematics in modulating the feedback responses.

Reviewer’s comment:

4) Line 47 “In this study we test to what degree optimal feedback control, as opposed to other control methods, can be used to model the visuomotor feedback responses directly.”

This study appears to only study various versions of optimal feedback control, eg classic finite-horizon, time to target adjusted, receding horizon, or infinite-horizon OFC.

Response:

Corrected.

Reviewer’s comment:

5) Line 77 “we devised an experimental paradigm where we offset the usual bell-shaped velocity profile in the aim to separate the effect of the times-to-target from the effect of kinematics on the visuomotor feedback responses.”

I question the use of the words “effect of kinematics” as a single term here and a couple other places in the manuscript. Without a qualifier, I tend to view kinematics as referring to the physical movement of the hand. But it overall is unclear whether this refers to the hand kinematics or the visual display kinematics with the given experiment. And I think here the author is referring to the combination of effects. This should be clarified and probably always explicitly stated in the manuscript since the discrepancies between hand and display are a critical part of the experiment.

Response:

This raises a good point. We now clarify that by kinematics we mean both cursor and hand kinematics together. In cases when we specifically talk about physical or visual kinematics we specify that accordingly. We went over the manuscript and removed any inconsistencies.

Reviewer’s comment:

Other comments:

Line 116 - It should be made clearer that this is referring to the forward hand velocity-with no influence of lateral hand velocity-so the forward distance traveled has a direct relationship to forward velocity. At least I assume that’s the case.

Response:

Corrected.

Reviewer’s comment:

Line 135 “was required to be similar to the baseline velocity profile” Is there a different between ideal trials and correct trials. For trials without the correct peak velocity and peak location, were no points scored and was there an error tone or is that just for overshoots?

Response:

Correct trials and ideal trials are synonyms. Currently we only use ideal trials to describe them. No points were scored if the peak velocity or location was out of the ideal range, or if the target was overshot. The error tone was only present in case of the overshoot, otherwise just the feedback was provided (Figure 1). All trials were used for analysis.

We have included this in the methods.

Reviewer’s comment:

Line 136 Feedback was always provided about this specific velocity modality. This is a vague reference to the preceding sentence, I’d explicitly say feedback was for the velocity modality that matched baseline. I’d also recommend explicitly stating the feedback could be in hand coordinates as I believe a reader might easily assume the feedback on a display would always be in cursor space as I did at first.

Response:

Corrected.

Reviewer’s comment:

Figure 1 legend. “Feedback was provided on the modality (cursor or hand) that matched the baseline.” Does this just mean the feedback was provided in the display at the location in the center of the forward axis where it occurred for baseline movements?

Response:

Yes. Added explanation.

Reviewer’s comment:

Line 144 y-coordinate is not defined, I assume the forward coordinate

Response:

Corrected.

Reviewer’s comment:

Line 153 - “The lateral hand position was constrained...” It should be clarified whether this refers to all trials or only probe trials. And then whether there was any difference for the mechanical channel also for the half of perturbation trials that required correction throughout the trial. If it is all trials, this section should not be placed here with probe trials but earlier.

Response:

This is only for the probe trials. Maintained perturbation trials were not constrained and required a lateral correction to successfully complete the trial. We have clarified this in the text.

Reviewer’s comment:

Line 438 and Figure 11a Can the authors clarify what is intended by this plot. I assume all points should fit the black line but it is ambiguous with the individual conditions/colors following a somewhat similar increase and decrease when looked at individually.

Response:

Our original figure 11A was not actually fit to the data and its purpose was to demonstrate similar qualitative features between data and model. During this revision, as we added additional quantitative analysis, we now fit this curve to the data and its fit is described in the Results. Specifically, the tuning curve demonstrated in Fig 11A accounts for similar amount of variance as the time-to-target OFC model, and more than the classical OFC. Further, we now also provide a similar fit to the data of Dimitriou et al. 2013 in Fig 11C
